# Low androgen levels induce ferroptosis of rat penile cavernous endothelial cells

**DOI:** 10.1093/sexmed/qfad043

**Published:** 2023-08-04

**Authors:** Hong-Xing Shi, Xin Zhao, Haifan Yang, Yong Cheng, Jun Jiang, Rui Jiang

**Affiliations:** Department of Urology, Affiliated Hospital of Southwest Medical University, Luzhou, Sichuan 646000, China; Department of Urology, Affiliated Hospital of Southwest Medical University, Luzhou, Sichuan 646000, China; Department of Urology, Affiliated Hospital of Southwest Medical University, Luzhou, Sichuan 646000, China; Department of Urology, Affiliated Hospital of Southwest Medical University, Luzhou, Sichuan 646000, China; Department of Thyroid Surgery, Affiliated Hospital of Southwest Medical University, Luzhou, Sichuan 646000, China; Department of Urology, Affiliated Hospital of Southwest Medical University, Luzhou, Sichuan 646000, China; Nephropathy Clinical Medical Research Center of Sichuan Province, Affiliated Hospital, Southwest Medical University, Taiping Road, Luzhou, Sichuan 646000, China

**Keywords:** androgen, ferroptosis, endothelial cells, NO

## Abstract

**Background:**

Endothelial dysfunction caused by low androgen levels in penile tissue can lead to erectile dysfunction. The exact mechanism of endothelial dysfunction has not been thoroughly studied.

**Objective:**

The study sought to verify whether low androgen levels induce ferroptosis of endothelial cells in rat penile tissue.

**Methods:**

Rat penile cavernous endothelial cells (CP-R133) were divided into a no-androgen group (Dihydrotestosterone (DHT): 0 nmol/L), very low-androgen group (DHT: 0.1 nmol/L), low-androgen group (DHT: 1 nmol/L), DHT = 10 nmol/L group, DHT (0 nmol/L) + ferrostatin-1 (Fer-1) group, DHT (0.1 nmol/L) + Fer-1 group, DHT (1 nmol/L) + Fer-1 group, DHT (10 nmol/L) + Fer-1 group. Cell viability, intracellular ferrous ion (Fe^2+^), malondialdehyde (MDA), GSH into oxidized glutathione (GSSG), reactive oxygen species (ROS), nitric oxide (NO), transferrin receptor 1 protein (TfR1), solute carrier family 7 member 11 (SLC7A11), glutathione peroxidase 4 (GPX4), acyl-CoA synthetase long-chain family member 4 (ACSL4), endothelial nitric oxide synthase (eNOS), and phospho-eNOS (p-eNOS) were detected.

**Outcomes:**

Low androgen levels could induce ferroptosis of rat penile cavernous endothelial cells in vivo by upregulating the expressions of TfR1 and ACSL4 and downregulating the expressions of SLC7A11 and GPX4.

**Results:**

Cell viability, the levels of glutathione (GSH), NO, SLC7A11, GPX4, and p-eNOS/eNOS in the DHT = 0 nmol/L group were lower than those in the other groups (*P* < .05). The levels of Fe^2+^, ROS, MDA, GSSG, TfR1, and ACSL4 in the DHT = 0 nmol/L group were higher than those in the other groups (*P* < .05). Cell viability and the levels of GSH, NO, SLC7A11, GPX4, and p-eNOS/eNOS in the DHT = 1 nmol/L group were lower than those in the DHT (1 nmol/L) + Fer-1 group, DHT = 10 nmol/L group, and DHT (10 nmol/L) + Fer-1 group (*P* < .05). The levels of Fe^2+^, ROS, MDA, GSSG, TfR1, and ACSL4 in the DHT = 1 nmol/L group were higher than those in the DHT (1 nmol/L) + Fer-1 group, DHT = 10 nmol/L group, and DHT (10 nmol/L) + Fer-1 group (*P* < .05).

**Clinical Implications:**

A ferroptosis inhibitor might be a novel drug for treating erectile dysfunction caused by low androgen level.

**Strengths and Limitations:**

The results of this study need to be further confirmed in in vitro and in human studies. Meanwhile, further investigation is needed to clarify whether low androgen levels affect ferroptosis of rat penile cavernous smooth muscle and nerve cells.

**Conclusion:**

Low androgen levels can induce ferroptosis of endothelial cells in rat penile tissue. Inhibition of ferroptosis can reverse endothelial dysfunction caused by low androgen levels.

## Introduction

Erectile dysfunction (ED) is the inability to reliably obtain and sustain a penile erection, which is necessary for sexual intercourse.^1^ ED mainly occurs in men over 40 years of age.^2^ One of the etiologies for ED is low testosterone levels. The prevalence of ED is greater in men who have androgen insufficiency than in common men.^3^ As the main medication used to treat ED, phosphodiesterase type 5 inhibitors achieved an effectiveness of <70%.^4^ It has been demonstrated that androgen replacement can enhance erectile performance in ED patients with low testosterone levels when a phosphodiesterase type 5 inhibitor is ineffective.^4^^,^^5^ However, the maintenance of low testosterone levels is essential for many ED patients who experience prostate cancer with androgen depletion therapy. Thus, the development of new therapeutic targets for ED patients should be explored in more detail.

In addition to arousing libido, androgen is primarily responsible for regulating appropriate erectile function and maintaining the integrity of penile cavernous vascular endothelial cells.^6^^,^^7^ Under low-androgen circumstances, penile cavernous tissue would produce an excess of reactive oxygen species (ROS), which cause endothelial dysfunction by affecting the integrity of penile cavernous vascular endothelial cells.^8^^,^^9^ Furthermore, low androgen levels have been linked to endothelial cell apoptosis and pyroptosis in penile cavernous tissue. This leads to ED by reducing the number of endothelial cells.^10^^,^^11^

Ferroptosis, a recently identified kind of regulated cell death, differs from apoptosis, pyroptosis, and other types of death. The characteristics of ferroptosis are iron dependency and ROS-induced lipid peroxidation.^12^^,^^13^ The main cause of ferroptosis is that iron ion metabolism disorder leads to excessive uptake of iron ions by cells and damages the antioxidant function, which caused an increase in the levels of ROS and the peroxidation of polyunsaturated fatty acids to produce lipid peroxides. Then, the cell lipid membrane structure was destroyed and the cells were dead.^14^ It has been reported that iron ion metabolism disorder, antioxidant system inhibition, and lipid peroxidation are the main mechanisms of ferroptosis.^15^ As the ferroptosis activating gene, *transferrin receptor 1 protein* (*TfR1*) could promote the absorption of the iron ion, resulting in a high concentration of iron ions in cells and inducing ferroptosis.^16^ Three major antioxidant systems have been found to be involved in the regulation of ferroptosis: system Xc^−^/glutathione (GSH)/glutathione peroxidase 4 (GPX4), ferroptosis suppressor protein 1 (FSP1)/coenzyme Q_10_ (CoQ_10_)/NADPH, and guanosine triphosphate cyclohydrolase 1 (GCH1)/dihydrofolate (DHFR)/tetrahydrobiopterin (BH_4_).^17^ System Xc^−^/GSH/GPX4 is the most important antioxidant system to regulate ferroptosis, and as a major component of cystine-glutamate antiporter (system Xc^−^), solute carrier family 7 member 11 (SLC7A11) is involved in the synthesis of GSH. GSH is an antioxidant that can effectively reduce the ROS level to prevent lipid peroxidation.^18^ GPX4 could convert GSH into oxidized glutathione (GSSG) and degraded lipid peroxides into lipoid alcohol to reduce cytotoxicity and avoid cell damage.^19^  *SLC7A11 *and *GPX4* have been described as the ferroptosis suppressor genes.^20^ In addition, FSP1 promotes the production of endogenous fat-solute antioxidant CoQ_10_ in the FSP1/CoQ_10_/NADPH system, which could prevent ferroptosis by inhibiting lipid peroxidation.^21^ In the GCH1/DHFR/BH_4_ system, as the rate-limiting enzyme in the synthesis of BH_4_, GCH1, and DHFR could produce a lipophilic antioxidant BH_4_ to prevent ferroptosis.^22^ As independent antioxidant systems, FSP1/CoQ_10_/NADPH and GCH1/DHFR/BH_4_ can cooperate with system Xc^−^/GSH/GPX4 to regulate ferroptosis in a GSH-independent manner. The production of lipid peroxides is one of the key steps in the occurrence of ferroptosis. When ROS level increased, polyunsaturated fatty acid was oxidized to lipid peroxides under acyl-CoA synthetase long-chain family member 4 (ACSL4). Lipid peroxides could be decomposed into toxic derivatives such as malondialdehyde (MDA).^23^ As a selective inhibitor of ferroptosis, Ferrostatin-1 (Fer-1) could maintain iron homeostasis by removing excessive ferrous ions, increase the GSH content by upregulating the expression of SLC7A11 and GPX4, and reduce the production of lipid peroxides by downregulating the expression of ACSL4, which could prevent ferroptosis.^24^

To date, ferroptosis has been researched in many systemic illnesses, such as cancer, heart failure, liver injury, and diabetes.^25–28^ After antioxidant activity in endothelial cells was compromised, the accumulation of ROS would trigger ferroptosis.^29^ It has been established that ROS level increased in penile cavernous tissue caused by low androgen levels is one of the mechanisms of ED.^8^^,^^9^ The ROS level increased regarded as an important process in the newly discovered ferroptosis pathway, and androgen receptor antagonists can induce ferroptosis in prostate cancer cells during androgen depletion therapy for prostate cancer patients.^30^ Based on these findings, it can be inferred that androgen levels may be related to ferroptosis. Can low androgen status affect ferroptosis of endothelial cells in the penile corpus cavernosum? This study aimed to investigate whether androgen levels can affect the ferroptosis suppressor genes (*SLC7A11* and *GPX4*) and the ferroptosis activating genes (*TfR1* and *ACSL4*) in rat penile cavernous endothelial cells. This may lay an important foundation for finding new approaches for improving erectile function in hypoandrogenism.

## Methods

### Cell grouping

Rat penile cavernous endothelial cells (CP-R133) (Procell) were evenly inoculated in culture flasks that contained EGM-2 endothelial cell culture medium (Lonza) and 5% fetal bovine serum (ScienCell). When the growth density of endothelial cells reached approximately 70% to 80%, endothelial cells were digested by trypsin cell digestive solution (Beyotime) and uniformly seeded in 6-well plates for culture. Those cells were divided into a no-androgen group (Dihydrotestosterone (DHT): 0 nmol/L), very low-androgen group (DHT: 0.1 nmol/L), low-androgen group (DHT: 1 nmol/L), DHT = 10 nmol/L group, DHT (0 nmol/L) + Fer-1 group, DHT (0.1 nmol/L) + Fer-1 group, DHT (1 nmol/L) + Fer-1 group, and DHT (10 nmol/L) + Fer-1 group.

### Cell viability

Endothelial cells were prepared as a single-cell suspension with a density of approximately 5 × 10^4^/mL and inoculated in a 96-well plate, each well was added by 100 μL cell suspension, and the cells grew adherently after culturing for 4 hours. Then, DHT and Fer-1 were used to divide endothelial cells into different groups. After culturing for 24 hours, 10 μL CCK-8 and 100 μL complete culture medium were added to wells. The cell viability was measured by a microplate reader after culturing for 1 hour.

### Intracellular ferrous ion concentration

The ferrous iron (Fe^2+^) concentration was detected by the iron assay kit (Abcam). The detection sample and standard were uniformly added to the wells and cultured at 37 °C for 30 minutes, and then a 100 μL iron probe was added to wells. The Fe^2+^ concentration was measured by a microplate reader after culturing for 1 hour protected from light.

### ROS level detected by flow cytometry (FCM)

The level of ROS was detected by the ROS assay kit (Beyotime). Serum-free medium was used to dilute DCFH-DA into a solution at a concentration of 10 μM. The diluted DCFH-DA solution was added to cells. The fluorescence intensity was detected by a flow cytometer (Beckman Coulter) using a 488-nm stimulated light wavelength and a 525-nm emission light wavelength after culturing for 30 minutes at 37 °C protected from light.

### TfR1, SLC7A11, GPX4, ACSL4, endothelial nitric oxide synthase, and phosphorylated endothelial nitric oxide synthase detected by Western blotting

Each group of CP-R133 cells was treated separately, the prechilled RIPA lysate (Beyotime) was used to digest cells on the ice surface for 30 minutes, and the cell suspension was centrifuged at a rate of 12 000 rpm for 15 minutes at 4 °C. A bicinchoninic acid assay kit (Beyotime) was used to detect the total protein concentration, and 5× loading buffer (Beyotime) was added to the supernatant. The mixture was denatured for 10 minutes at 100 °C and stored at −20 °C. The proteins in each histone sample were separated by electrophoresis on the gel. After the proteins transferred to a polyvinylidene fluoride membrane, they were blocked with 5% skim milk for 1 hour, and then the membrane and primary antibody were placed together overnight at 4°C. The primary antibodies included mouse anti-TfR1 antibody (1:5000; Abcam), rabbit anti-SLC7A11 antibody (1:1000; Abcam), rabbit anti-GPX4 antibody (1:1000; Abcam), rabbit anti-ACSL4 antibody (1:10 000; Abcam), rabbit anti-endothelial nitric oxide synthase (eNOS) antibody (1:1000; Abcam), rabbit anti-phospho-eNOS (p-eNOS) antibody (1:1000; Abcam), and mouse anti-GAPDH antibody (1:20 000; Proteintech Group). The membranes were incubated with a goat anti-rabbit/mouse secondary antibody (1:2000; Beyotime) for 1 hour at normal temperature. Finally, ECL luminescence solution (ShareBio) was added to obtain a protein band image, and Image software Quantity One 4.6 (Bio-Rad Laboratories) was used to analyze the protein content.

### Concentrations of MDA, GSH, GSSG, and nitric oxide

The concentrations of MDA, GSH, GSSG, and nitric oxide (NO) were detected following the respective reagent instructions of the MDA, GSH, GSSG assay kit (Nanjing Jiancheng Bioengineering Institute), and NO assay kit (Beyotime). The optical density was determined by a microplate reader.

### Statistical analysis

The experimental data are expressed as the mean ± SD, and the data were analyzed by GraphPad Prism 8.0 software (GraphPad Software). The statistical method used was 1-way analysis of variance and linear correlation analysis, and *P* < .05 was considered statistically significant.

## Result

### Cell viability in each group

**Figure 1:**
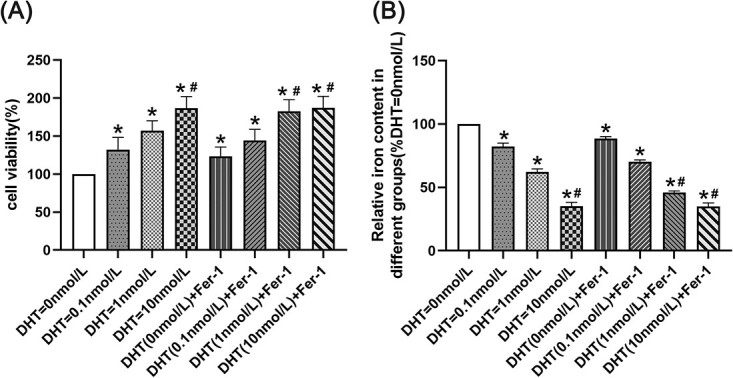
Cell viability and iron content in each group. (A) Cell viability was detected by CCK-8. (B) Iron content was detected by the iron assay kit. ^*^P < .05 vs the no-androgen group; ^#^*P* < .05 vs the low-androgen group.

Cell viability in the DHT = 0 nmol/L group was lower than that in the other groups (*P* < .05). Cell viability in the DHT = 1 nmol/L group was lower than that in the DHT (1 nmol/L) + Fer-1 group, DHT = 10 nmol/L group, and DHT (10 nmol/L) + Fer-1 group (*P* < .05). Cell viability in the DHT = 10 nmol/L group showed no significant difference compared with the DHT (10 nmol/L) + Fer-1 group (Figure 1).

### Fe ^2+^ concentration in endothelial cells

The Fe^2+^ concentration in the DHT = 0 nmol/L group was higher than that in the other groups (*P* < .05). The Fe^2+^ concentration in the DHT = 1 nmol/L group was higher than that in the DHT (1 nmol/L) + Fer-1 group, DHT = 10 nmol/L group, and DHT (10 nmol/L) + Fer-1 group (*P* < .05). The Fe^2+^ concentration in the DHT = 10 nmol/L group showed no significant difference compared with the DHT (10 nmol/L) + Fer-1 group (Figure 1).

### ROS level in endothelial cells

**Figure 2:**
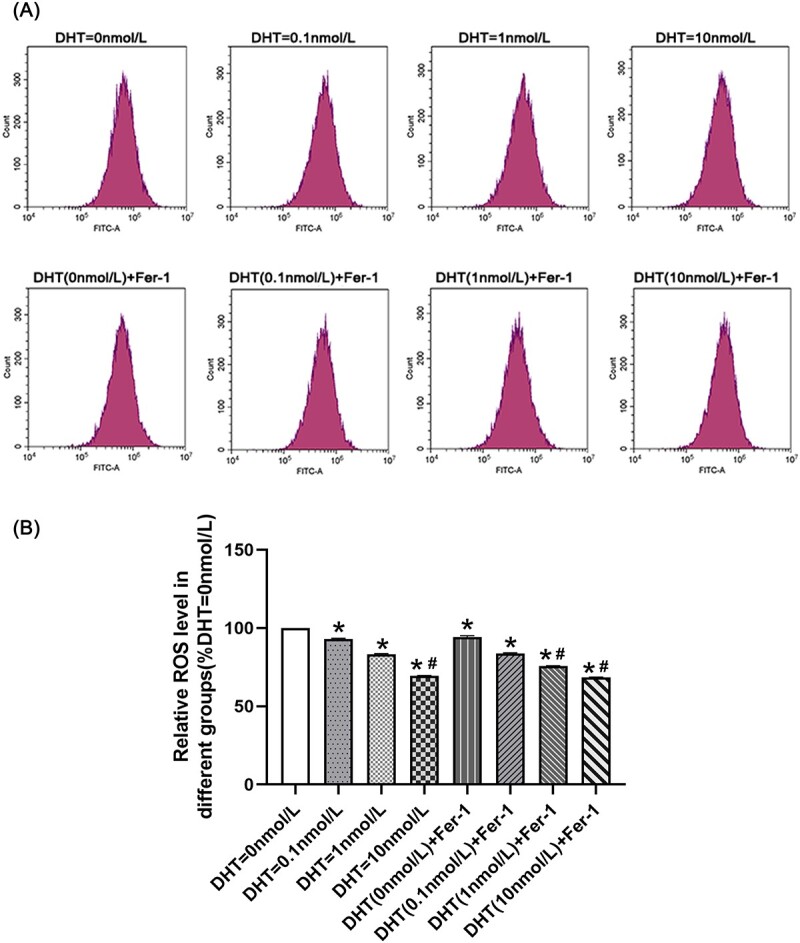
The generation of reactive oxygen species (ROS) was determined in each group. (A) Histogram was used to present ROS levels. (B) Data are presented as the relative ROS levels in each group. ^*^P < .05 vs the no-androgen group; ^#^*P* < .05 vs the low-androgen group.

The ROS level in the DHT = 0 nmol/L group was higher than that in the other groups (*P* < .05). The ROS level in the DHT = 1 nmol/L group was higher than that in the DHT (1 nmol/L) + Fer-1 group, DHT = 10 nmol/L group, and DHT (10 nmol/L) + Fer-1 group (*P* < .05). The ROS level in the DHT = 10 nmol/L group showed no significant difference compared with the DHT (10 nmol/L) + Fer-1 group (Figure 2).

### Western blotting

**Figure 3:**
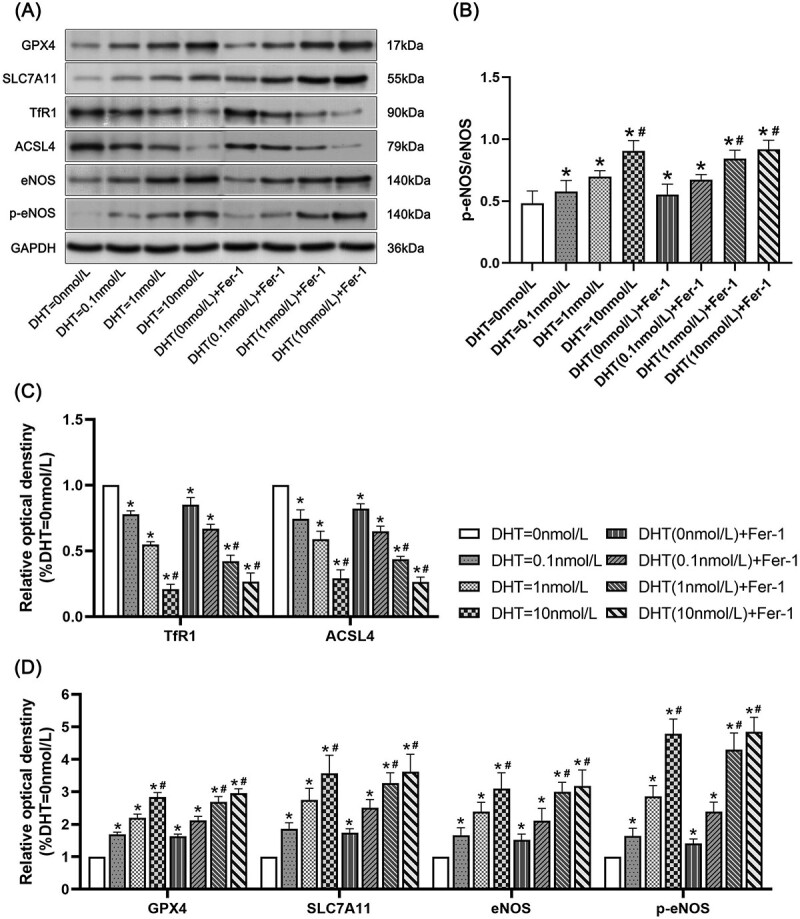
The expressions of GPX4, x-CT, TfR1, ACSL4, endothelial nitric oxide synthase (e-NOS), and phospho-eNOS (p-eNOS) in rat penile cavernous endothelial cells. (A) The analyses of GPX4, x-CT, TfR1, ACSL4, e-NOS and p-eNOS were by Western blotting in each group. (B) Data are presented as the relative density values of GPX4, x-CT, TfR1, ACSL4, e-NOS, and p-eNOS. (C) Data are presented as the ratio of p-eNOS/eNOS. ^*^P < .05 vs the no-androgen group; ^#^*P* < .05 vs the low-androgen group.

The expressions of GPX4, SLC7A11, endothelial nitric oxide synthase (eNOS), and phospho-eNOS (p-eNOS) in the DHT = 0 nmol/L group were lower than those in the other groups (*P* < .05). The expressions of GPX4, SLC7A11, eNOS, and p-eNOS in the DHT = 1 nmol/L group were lower than those in the DHT (1 nmol/L) + Fer-1 group, DHT = 10 nmol/L group, and DHT (10 nmol/L) + Fer-1 group (*P* < .05). The expressions of GPX4, SLC7A11, eNOS, and p-eNOS in the DHT = 10 nmol/L group showed no significant difference compared with the DHT (10 nmol/L) + Fer-1 group. The expressions of TfR1 and ACSL4 in the DHT = 0 nmol/L group were higher than those in the other groups (*P* < .05). The expressions of TfR1 and ACSL4 in the DHT = 1 nmol/L group were higher than those in the DHT (1 nmol/L) + Fer-1 group, DHT = 10 nmol/L group, and DHT (10 nmol/L) + Fer-1 group (*P* < .05). The expressions of TfR1 and ACSL4 in the DHT = 10 nmol/L group showed no significant difference compared with the DHT (10 nmol/L) + Fer-1 group (Figure 3).

### MDA, GSH, GSSG, and NO content in endothelial cells

**Figure 4:**
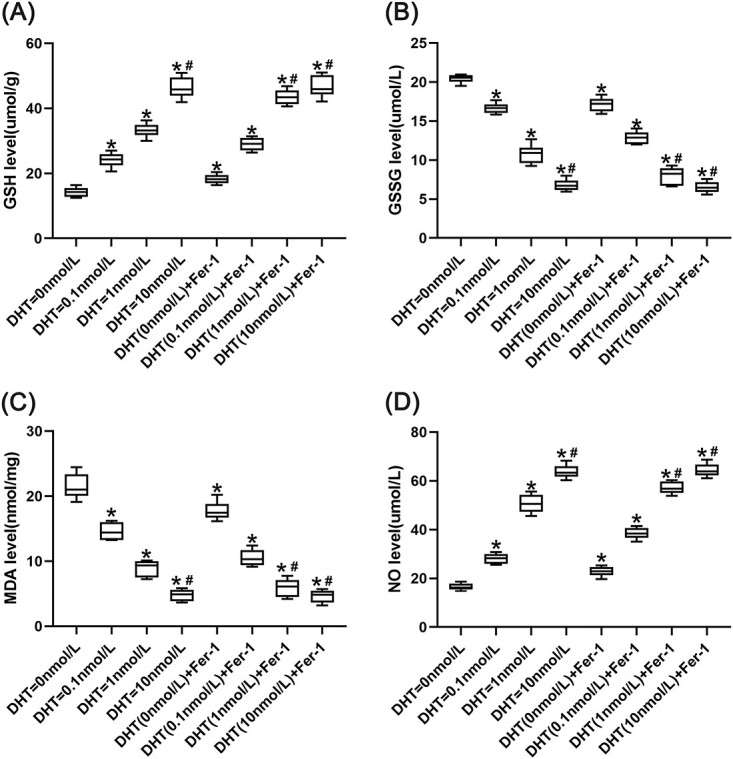
The concentrations of GSH, oxidized GSH (GSSG), malondialdehyde (MDA), and nitric oxide (NO) were detected in each group. (A) Data are presented as the concentration of GSH in each group; (B) data are presented as the concentration of GSSG in each group; (C) data are presented as the concentration of MDA in each group; (D) data are presented as the concentration of NO in each group. ^*^P < .05 vs the no-androgen group; ^#^*P* < .05 vs the low-androgen group.

The concentrations of GSH and NO in the DHT = 0 nmol/L group were lower than those in the other groups (*P* < .05). The concentrations of GSH and NO in the DHT = 1 nmol/L group were lower than those in the DHT (1 nmol/L) + Fer-1 group, DHT = 10 nmol/L group, and DHT (10 nmol/L) + Fer-1 group (*P* < .05). The concentrations of GSH and NO in the DHT = 10 nmol/L group showed no significant difference compared with the DHT (10 nmol/L) + Fer-1 group. The concentrations of MDA and GSSG in the DHT = 0 nmol/L group were higher than those in the other groups (*P* < .05). The concentrations of MDA and GSSG in the DHT = 1 nmol/L group were higher than those in the DHT (1 nmol/L) + Fer-1 group, DHT = 10 nmol/L group, and DHT (10 nmol/L) + Fer-1 group (*P* < .05). The concentrations of MDA and GSSG in the DHT = 10 nmol/L group showed no significant difference compared with the DHT (10 nmol/L) + Fer-1 group (Table 1, Figure 4).

**Table 1:** The levels of GSH, GSSG, MDA, and NO in rat penile cavernous endothelial cells in each group.

Group (n = 8)	GSH (μmol/g)	GSSG (μmol/L)	MDA (nmol/mg)	NO (μmol/L)
DHT = 0 nmol/L	14.26 ± 1.47	20.45 ± 0.56	21.49 ± 1.96	16.58 ± 1.37
DHT = 0.1 nmol/L	24.14 ± 2.19^a^	16.65 ± 0.65^a^	14.57 ± 1.30^a^	28.14 ± 2.02^a^
DHT = 1 nmol/L	33.26 ± 2.14^a^	10.79 ± 1.20^a^	8.93 ± 1.28^a^	50.67 ± 3.80^a^
DHT = 10 nmol/L	46.34 ± 3.24^a^^,^^b^	6.80 ± 0.74^a^^,^^b^	4.78 ± 0.86^a^^,^^b^	63.80 ± 2.74^a^^,^^b^
DHT (0 nmol/L) + Fer-1	18.29 ± 1.41^a^	17.13 ± 0.88^a^	17.75 ± 1.41^a^	22.84 ± 1.99^a^
DHT (0.1 nmol/L) + Fer-1	29.00 ± 1.96^a^	12.87 ± 0.79^a^	10.53 ± 1.23^a^	38.50 ± 2.37^a^
DHT (1 nmol/L) + Fer-1	43.47 ± 2.28^a^^,^^b^	7.99 ± 1.13^a^^,^^b^	5.93 ± 1.35^a^^,^^b^	57.13 ± 2.43^a^^,^^b^
DHT (10 nmol/L) + Fer-1	46.65 ± 3.32^a^^,^^b^	6.51 ± 0.76^a^^,^^b^	4.63 ± 0.95^a^^,^^b^	64.34 ± 2.70^a^^,^^b^

Values are mean ± SD.

Abbreviations: GSSG, oxidized glutathione; MDA, malondialdehyde; NO, nitric oxide.

^a^ *P* < .05 vs no-androgen group.

^b^ *P* < .05 vs low-androgen group.

## Discussion

In rat penile tissue, the function of endothelial cells is highly associated with androgen levels.^31^^,^^32^ Several existing studies have shown that low androgen levels induced oxidative stress and increased ROS level could promote endothelial dysfunction,^8^^,^^9^ and androgen supplementation therapy can effectively reduce ROS level, resulting in improving erectile function by antioxidant effects in castrated rats.^9^ Oxidative stress and increased levels of ROS are involved in the occurrence of ferroptosis. To explore the relationship between androgens and ferroptosis of endothelial cells, we chose 0 nM, 0.1 nM, 1 nM, and 10 nM DHT to culture endothelial cells.^25^ Clinically, prostate cancer patients need to maintain low testosterone levels during androgen deprivation therapy. Typically, their serum testosterone levels are below 20 ng/dL (1 nM).^33^ To further verify the occurrence of ferroptosis in rat penile cavernous endothelial cells under low-androgen conditions, Fer-1, as a ferroptosis inhibitor, was selected to inhibit ferroptosis in the low-androgen group.

The system Xc^−^/GSH/GPX4, as the first antioxidant system found to be involved in the regulation of ferroptosis, has been widely studied, while the FSP1/CoQ_10_/NADPH and GCH1/DHFR/BH_4_ systems have been less studied. Thus, we mainly examined the expressions of system Xc^−^/GSH/GPX4 and other ferroptosis-related indicators in each group. As a result, with the 0 to 10 nM concentration of DHT, cell viability and the levels of p-eNOS/eNOS and NO were positively correlated with the DHT concentration. This indicated that DHT is an important condition for maintaining the survival and function of rat penile cavernous endothelial cells. These outcomes showed no difference with previous studies.^32^ The expression of TfR1 and Fe^2+^ concentration were negatively correlated with DHT concentration, suggesting that DHT could reduce the iron load by inhibiting the expression of *TfR1*, which is one of the ferroptosis activating genes. DHT concentration was positively correlated with the levels of GSH, SLC7A11, and GPX4 and negatively correlated with the levels of GSSG and ROS. These suggested that DHT could improve the antioxidant capacity of system Xc^−^/GSH/GPX4 and reduce ROS formation by upregulating the expressions of *SLC7A11* and *GPX4*, which are the ferroptosis suppressor genes. The expressions of ACSL4 and MDA concentrations were negatively correlated with DHT concentration, suggesting that DHT could reduce the production of cytotoxic MDA to prevent ferroptosis by downregulating the expression of ACSL4. Based on these findings, it was indicated that biochemical processes related to ferroptosis in rat penile cavernous endothelial cells are activated after cells were cultured in low androgen levels (0 nM, 0.1 nM, 1 nM). The processes induced by the iron ion metabolism load increased, antioxidant function of system Xc^−^/GSH/GPX4 was impaired, and ROS and lipid peroxidation increased, which finally resulting in decreased the levels of p-eNOS/eNOS and NO in vascular endothelial cells. It has been found that high glucose could induce ferroptosis of rat penile cavernous smooth muscle cells by upregulating the expression of ACSL4 and downregulating the expression of SLC7A11 and GPX4.^34^ However, the effect of high glucose on ferroptosis of rat penile cavernous endothelial cells has not been reported. In our study, we found that the expressions of SLC7A11 and GPX4 decreased and the expressions of TfR1 and ACSL4 increased in rat penile cavernous endothelial cells under low androgen levels, which was consistent with the molecular mechanism of ferroptosis in rat penile cavernous smooth muscle cells caused by high glucose. Because ferroptosis involves multiple signal transduction pathways, it may be difficult to effectively inhibit ferroptosis by gene knockout or single gene engineering methods. Therefore, Fer-1 was used to inhibit ferroptosis. After being treated by Fer-1, cell viability and the levels of GSH, NO, the ferroptosis suppressor genes (SLC7A11 and GPX4), and p-eNOS/eNOS were increased and the levels of Fe^2+^, ROS, MDA, GSSG, TfR1, and ACSL4 were decreased in the endothelial cells treated with low DHT levels. This indicates that the injured endothelial cells under low-androgen conditions could be reduced at least partially by the inhibition of ferroptosis.

To our knowledge, this study is the first to report that low androgen levels could induce ferroptosis of rat penile cavernous endothelial cells in vivo by upregulating the expressions of TfR1 and ACSL4 and downregulating the expressions of SLC7A11 and GPX4 (Supplementary Figure 5). Fer-1 could improve endothelial function under low androgen levels by inhibiting ferroptosis. This could be a new way to treat ED patients caused by low androgen levels. Nevertheless, the results of this study need to be further confirmed in in vitro and in human studies. Meanwhile, further investigation is needed to clarify whether low androgen levels affect ferroptosis of rat penile cavernous smooth muscle cells and nerve cells, as well as its relationship with changes in mitochondrial ultrastructures. The use of a ferroptosis activator as a positive control may further clarify the relationship between the activity of penile corpus cavernosum endothelial cells and ferroptosis. Further research is needed to investigate whether there is a difference in the mechanism of cell ferroptosis induced by low androgen levels and lipopolysaccharide and oxidized low-density lipoprotein. Additionally, this study confirmed that low androgen levels induce ferroptosis of rat penile cavernous endothelial cells by inhibiting the system Xc^−^/GSH/GPX4 pathway. However, whether there are other pathways (such as FSP1/CoQ_10_/NADPH, GCH1/DHFR/BH_4_) involved in ferroptosis caused by low androgen levels that need to be further explored, too.

## Supplementary Material

Supplementary_material_qfad043

A003-1_MDA_Assay_Kit_Instruction_qfad043

A006-2_GSH_Assay_Kit_Instruction_qfad043

A012-1_NO_Nitrate_Reductase_Assay_Kit_Instruction_qfad043

A061-1_Total-GSH_Assay_Kit_Instruction_qfad043

## Data Availability

Data will be made available on request.
