# Supplementary material for: Low androgen levels induce ferroptosis of rat penile cavernous endothelial cells
Source: Sex Med. 2023 Aug 4;11(4):qfad043. doi: 10.1093/sexmed/qfad043 (PMC10401903; doi:10.1093/sexmed/qfad043)
Supplement: Supplementary_material_qfad043 [file supplementary_material_qfad043.docx]

Supplementary material：

The red fluorescence in this image is represented the positive expression of CD31 in endothelial cells. The rate of CD31-positive cells > 90%.


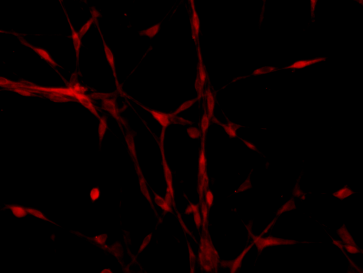

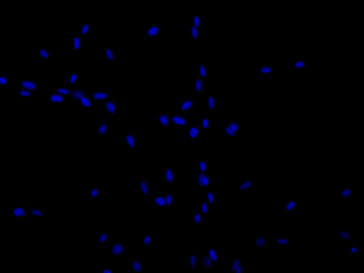

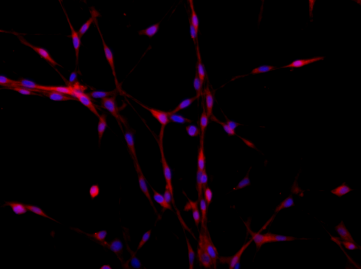


CD31 DAPI Merge


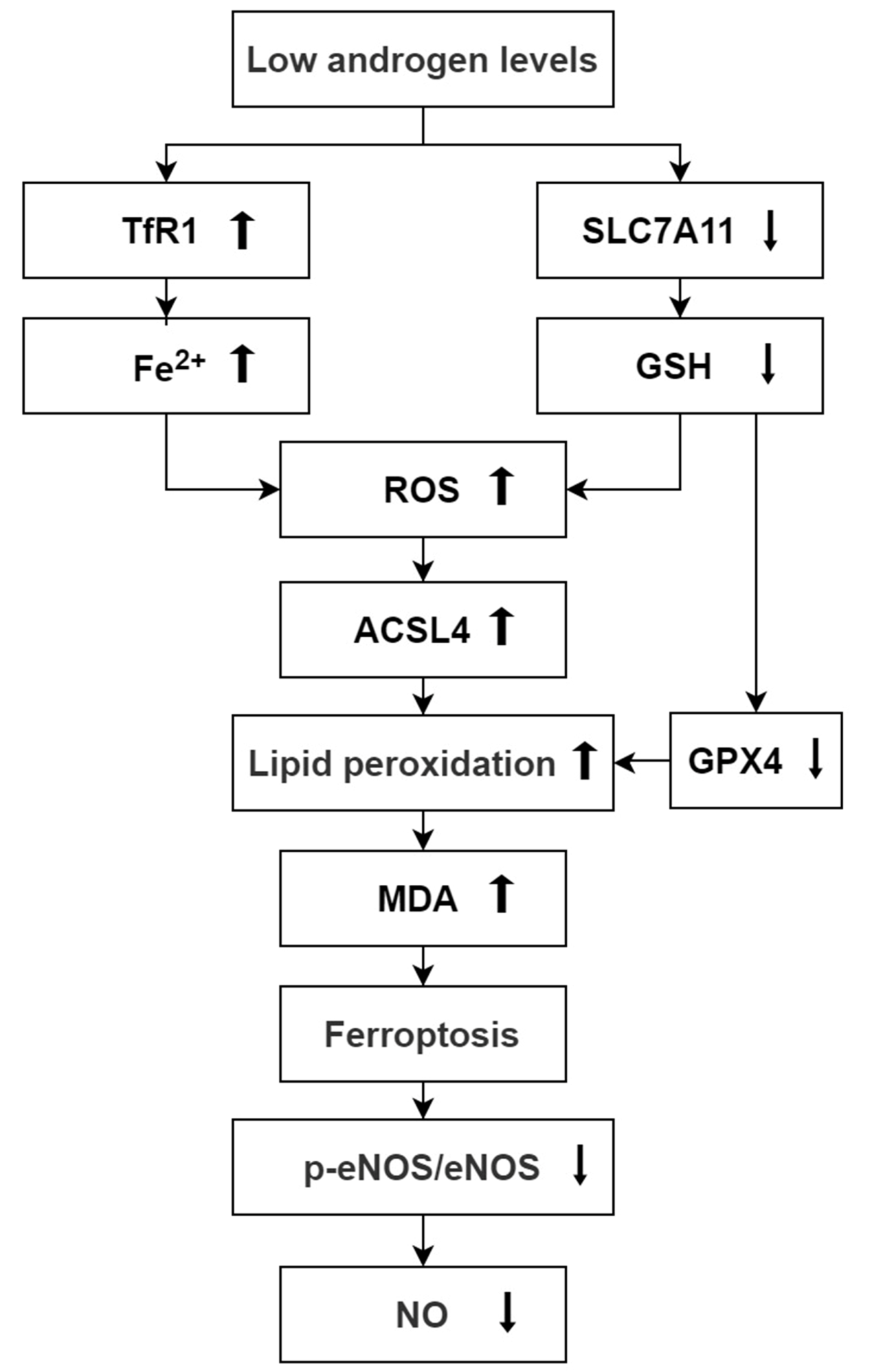


**Figure 5** Low androgen levels impaired endothelial function by inducing ferroptosis of rat penile cavernous endothelial cells. Low androgen levels increased ROS level resulting from high Fe^2+^ concentration caused by upregulating the expression of TfR1 and low GSH concentration caused by downregulating the expression of SLC7A11. Low androgen levels induced ferroptosis resulting from increasing the production of lipid peroxides caused by upregulating the expression of ACSL4 and high ROS level. Low androgen levels decreased the expression of GPX4 promoting lipid peroxidation. Ferroptosis decreased the levels of p-eNOS/eNOS and NO resulting from endothelial dysfunction. TfR1, transferrin receptor 1 protein; SLC7A11, solute carrier family 7 member 11; GPX4, glutathione peroxidase 4; ACSL4, acyl-CoA synthetase long-chain family member 4; eNOS, endothelial nitric oxide synthase; p-eNOS, phospho-eNOS; ROS, reactive oxygen species; GSH, glutathione; MDA, malondialdehyde; NO, nitric oxide.
