# Supplementary material for: Low androgen levels induce ferroptosis of rat penile cavernous endothelial cells
Source: Sex Med. 2023 Aug 4;11(4):qfad043. doi: 10.1093/sexmed/qfad043 (PMC10401903; doi:10.1093/sexmed/qfad043)
Supplement: A003-1_MDA_Assay_Kit_Instruction_qfad043 [file a003-1_mda_assay_kit_instruction_qfad043.doc]

**Malondialdehyde（MDA）Assay Kit Instruction**

**(TBA Method)**

**Serial No: A003-1 Pack：50T/48S 100T/96S**

This kit is improved by many domestic & abroad methods, it is a microscale, convenient, accurate, unharmful measuring method. This assay can reflect lipid peroxide amount of cellular level and subcellular in extracellular fluids such as blood serum (or plasma) and milk, etc., various kinds of animal cells and plant cells such as erythrocytes, leucocytes, cultured cells, etc.

This kit can be used for laboratory research only.

**1.MDA assay significance:**

Organisms generate oxygen radicals by enzyme system and non-enzyme system, oxygen radicals will attack polyunsaturated fatty acid (PUFA) to initiate lipid peroxidation, as result, lipid peroxides such as aldehyde group (MDA), ketone group, hydroxyl group, carbonyl group, hydroperoxy radical or endoperoxy radical, and new oxygen radical, etc. Lipid peroxidation not only convert active oxygen to active chemicals (nonradical lipid decomposed product) but also amplify effect of active oxygen by chain reaction and branched chain reaction. Thus a primary active oxygen can lead to many lipid decomposed products, some of them are unharmful but others can cause cellular dysmetabolism and dysfunction or even cell death. Oxygen radicals cause cell damage by not only PUFA peroxidation but also lipid hydroperoxide decomposition products. As result, MDA content always reflects lipid peroxidation level in vivo, it can also reflect cell damage level indirectly.

Generally, MDA assay can go with SOD assay, SOD activity reflects oxygen radical removing abilility in vivo indirectly, MDA content reflects severity level of free radical attacking on body cells. Analysis on account of SOD & MDA results is helpful for developments of medical science, biology, pharmacology, industry and agriculture.

**2.MDA assay principle:**

MDA in lipid hydroperoxide decomposition products can condensate with thibabituric acid (TBA)* to produce red compounds which has absorption peak at 532nm.

***** Reaction substrate is thibabituric acid (TBA), so this method is named as “TBA method”.

**3. Required equipments and reagents**

● An spectrophotometer capable of measuring absorbance at 532nm

● Thermostatic water bath or air bath capable of controlling temperature at 95℃

● Desk centrifuge

● Glacial acetic acid (analytical pure, CH3COOH≥99.5%)

**4. Reagent composition & preparation:**

1. **100T/96S assay kit:**

**Reagent 1:** Solution, 20ml×1 bottle, can be stored at room temperature. (It may coagulate in cold days, so before use, please warm this bottle in water bath in order to increase dissolving rate. When solution becomes limpid, then you can use it for assay.)

**Reagent 2:** Solution, 12ml×1 bottle. When use, add 340ml double distilled water in each bottle. Can be stored at 4℃. **Note: Never drop on your skin**.

**Reagent 3:** Powder×1 vial. When use, add powder in 60ml 90℃～100℃ hot double distilled water, dissolve sufficiently (you can heat properly during dissolving), add double distilled water until volume reaches to 60ml, add 60ml glacial acetic acid*, mix sufficiently, prepared reagent can be stored by cold preservation away from light (

**Standard:** 10 nmol/ml tetraethoxypropane, 5ml×1 bottle, can be stored at 4℃.

**This kit can be stored by cold preservation for at least 1 year.**

**(2) 50T/48S assay kit:**

**Reagent 1:** Solution, 10ml×1 bottle, can be stored at room temperature. (It may coagulate in cold days, so before use, please warm this bottle in water bath in order to increase dissolving rate. When solution becomes limpid, then you can use it for assay.)

**Reagent 2:** Solution, 6ml×1 bottle. When use, add 170ml double distilled water in each bottle. Can be stored at 4℃. **Note: Never drop on your skin**.

**Reagent 3:** Powder×1 vial. When use, add powder in 30ml 90℃～100℃ hot double distilled water, dissolve sufficiently (you can heat properly during dissolving), add double distilled water until volume reaches to 30ml, add 30ml glacial acetic acid*, mix sufficiently, prepared reagent can be stored by cold preservation away from light.

**Standard:** 10 nmol/ml tetraethoxypropane, 5ml×1 bottle, can be stored at 4℃.

This kit can be stored by cold preservation for at least 1 year.

**5. Normal Operation procedure:**

**(1) Normal operation table:**

|  | **Blank tube** | **Standard tube** | **Sample tube** | **Contrast tube**** |
| --- | --- | --- | --- | --- |
| 10 nmol/ml standard (ml) |  | a* |  |  |
| Dehydrated alcohol（ml） | a* |  |  |  |
| Sample to assay（ml） |  |  | a* | a* |
| Reagent 1 (ml) | a* | a* | a* | a* |
| Mix sufficiently by shaking test-tube stand | | | | |
| Reagent 2 (ml) | 3 | 3 | 3 | 3 |
| Reagent 3 (ml) | 1 | 1 | 1 |  |
| 50% glacial acetic acid（ml） |  |  |  | 1 |
| Mix sufficiently by vortex, seal test tube by refreshing film, pierce a small hole by needle, place in 95℃ water bath for 40 minutes, cool in tap water, centrifugate at 3500~4000rpm for 10 minutes.(If you centrifugate at low speed (<3000rpm), then please extend time in order to make sedimentation complete). Take supernatant***, transfer in cuvettes of 1cm light path, measure absorbances of all tubes at 532nm (adjust zero by double distilled water). | | | | |

**a* reflects volumes of sample, standard, dehydrated alcohol, Reagent 1, these four volumes should be equal to each other.** For example, if you take 0.1ml sample, then you should also take 0.1ml standard, dehydrated alcohol, Reagent 1; if you take 0.2ml sample, then you should also take 0.2ml standard, dehydrated alcohol, Reagent 1. Absorbance reflects direct proportion with sample volume, so result won’t be disturbed.

****Generally, it only needs to make 1~2 standard tubes, blank tubes, contrast tubes. If there is no haemolysis or lipidemia in samples, then you can skip contrast tube, use blank tube to instead.**

*******Use micropipet to transfer supernatant in cuvette, please avoid transfer by pouring or sediments may enter cuvette and disturb absorbance.

**(2) Referenced sample volume:**

● Blood serum (or plasma): 0.1~0.2ml

● Low density lipoprotein: 0.1~0.2ml

● Edible oil: 0.03ml

● Liver tissue, cardiac muscle, muscular tissue, spiral algae: 0.1~0.2ml 5% or 10% homogenate

**(3) Normal referenced standard absorbances:**

When standard volume is 0.1ml,

OD_Standard_ – OD_Blank_ = 0.065~0.070

When standard volume is 0.2ml,

OD_Standard_ – OD_Blank_ = 0.130~0.140

**(4)** **When you use normal operation procedure or convenient operation procedure, if OD_Sample_ is too low, then you can extend water bath period from 40 minutes to 80 minutes. But if you do like this, then please extend all MDA assays’ water bath period to 80 minutes in order to avoid difference between batches.**

**(5) Formulas:**

**①** **Blood serum (or plasma) MDA assay:**


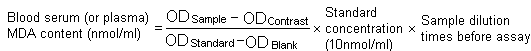


**②** **Tissue MDA assay:**


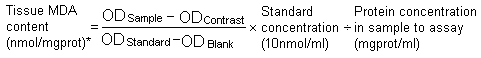


* nmol/mgprot means nanomole per protein in milligram

1. **Examples:**

① Take 0.1ml blood serum without dilution to measure MDA content, in results, OD_Sample_ is 0.036, OD_Standard_ is 0.076, OD_Contrast_ is 0.006 (use blank tube to instead contrast tube). Calculate as follows:


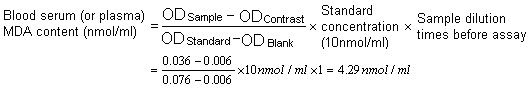


② Take 0.1ml yolk, add 0.9ml dehydrated alcohol, mix sufficiently, extract for 3 minutes, centrifugate at 4000rpm for 10 minutes, take 0.2ml supernatant to measure MDA content, in results, OD_Sample_ is 0.198, OD_Standard_ is 0.151, OD_Contrast_ is 0.009, (use blank tube to instead contrast tube). Calculate as follows:


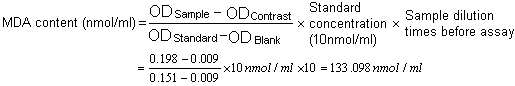


③ Take 0.1ml 10% rat liver tissue to measure MDA content, in results, OD_Sample_ is 0.214, OD_Standard_ is 0.079, OD_Contrast_ is 0.009, (use blank tube to instead contrast tube), protein concentration in 10% rat liver tissue homogenate is 8.5mgprot/ml. Calculate as follows:


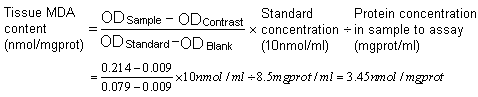


④ Take 0.1ml 10% carp brain tissue homogenate to measure MDA content, in results, in results, OD_Sample_ is 0.314, OD_Standard_ is 0.076, OD_Contrast_ is 0.006, (use blank tube to instead contrast tube), protein concentration in 10% carp brain tissue homogenate is 5.0788mgprot/ml. Calculate as follows:


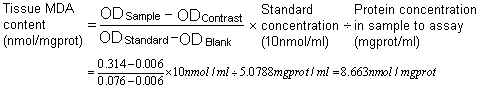


⑤ Take 0.1ml 20% earthworm tissue homogenate to measure MDA content, in results, in results, OD_Sample_ is 0.082, OD_Standard_ is 0.074, OD_Contrast_ is 0.005, OD_Blank_ is 0.025, protein concentration in 20% earthworm tissue homogenate is 10.3145 mgprot/ml. Calculate as follows:


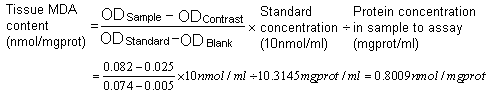


⑥ Take 0.2ml 10% rice leaf homogenate to measure MDA content, in results, in results, OD_Sample_ is 0.077, OD_Standard_ is 0.140, OD_Contrast_ is 0.003, (use blank tube to instead contrast tube), protein concentration in 20% earthworm tissue homogenate is 3.2729 mgprot/ml. Calculate as follows:


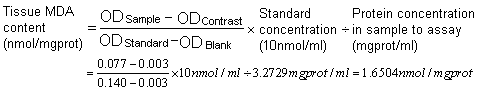


**6. Convenient operation procedure:**

**If number of samples is huge, then you can use convenient operation procedure.**

**(1) Mixed reagent preparation (volume ratio):**

**Working solution 1 preparation:**

Reagent 1: Reagent 2: Reagent 3 = a*:3:1, consider solution volume according to you need, this solution should be used at the same day of preparation.

**Working solution 2 preparation:**

Reagent 1: Reagent 2: 50% glacial acetic acid = a*:3:1, consider solution volume according to you need, this solution should be used at the same day of preparation.

1. **Convenient operation table:**

|  | Blank tube | Standard tube | Sample tube | Contrast tube** |
| --- | --- | --- | --- | --- |
| 10 nmol/m standard（ml） |  | a* |  |  |
| Dehydrated alcohol（ml） | a* |  |  |  |
| Sample to assay（ml） |  |  | a * | a* |
| Working solution I （ml） | 4ml | 4ml | 4ml |  |
| Working solution II (ml) |  |  |  | 4ml |
| Mix sufficiently by vortex, seal test tube by refreshing film, pierce a small hole by needle, place in 95℃ water bath for 40 minutes, cool in tap water, centrifugate at 3500~4000rpm for 10 minutes.(If you centrifugate at low speed (<3000rpm), then please extend time in order to make sedimentation complete). Take supernatant***, transfer in cuvettes of 1cm light path, measure absorbances of all tubes at 532nm (adjust zero by double distilled water). | | | | |

**a* reflects volumes of sample, standard, dehydrated alcohol, Reagent 1, these four volumes should be equal to each other.** For example, if you take 0.1ml sample, then you should also take 0.1ml standard, dehydrated alcohol, Reagent 1; if you take 0.2ml sample, then you should also take 0.2ml standard, dehydrated alcohol, Reagent 1. Absorbance reflects direct proportion with sample volume, so result won’t be disturbed.

**** You can skip contrast tube, use blank tube to instead.**

*******Use micropipet to transfer supernatant in cuvette, please avoid transfer by pouring or sediments may enter cuvette and disturb absorbance.

**Note1:** Normal method and convenient method apply to samples of human, aninmals & plants (include serum, animal & plant tissue, body fluid, cells & cell culture fluid, etc.).

**Note2:** When you use normal operation procedure or convenient operation procedure, if ODSample is too low, then you can extend water bath period from 40 minutes to 80 minutes. But if you do like this, then please extend all MDA assays’ water bath period to 80 minutes in order to avoid difference between batches.

**(3) Formulas:**

**①** **Blood serum (or plasma) MDA assay:**


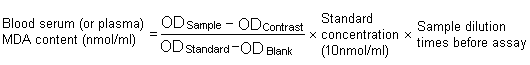


**②** **Tissue MDA assay:**


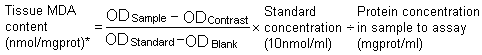


**(4) Examples:**

① Take 0.1ml undiluted blood serum to measure MDA content, in results, OD_Sample_ is 0.041, OD_Standard_ is 0.080, OD_Contrast_ is 0.006 (use blank tube to instead contrast tube). Calculate as follows:


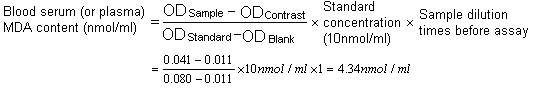


② Take 0.1ml 10% rat liver tissue homogenate to measure MDA content, in results, OD_Sample_ is 0.204, OD_Standard_ is 0.082, OD_Contrast_ is 0.013 (use blank tube to instead contrast tube), protein concentration in 10% rat liver tissue homogenate is 8.1mgprot/ml. Calculate as follows:


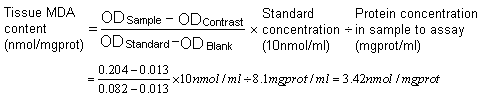


**7. Microscale operation procedure:**

**You can use microscale operation method for low MDA containing or smallscale samples such as mouse blood serum (or plasma), child blood serum (or plasma), mouse lung tissue, skin tissue, kidney tissue, retina, cells and cell culture fluid, etc:**

1. **Sample pretreatment follows tissue homogenate treatment in Experimental Methodology, make 10% or 5% homogenate. But please remember that it is better NOT to centrifugate because sample (such as lyzed cultured cells) is small scaled. Please take sample immediately after shaking.**
2. **Reagent composition & preparation:**

**① 100T/96S assay kit:**

**Reagent 1:** Solution, 20ml×1 bottle, can be stored at room temperature. (It may coagulate in cold days, so before use, please warm this bottle in water bath in order to increasing dissolving rate. When solution becomes limpid, then you can use it for assay.)

**Reagent 2:** Solution, 12ml×1 bottle. When use, add 340ml double distilled water in each bottle. Can be stored at 4℃. **Note: Never drop on your skin**.

**Reagent 3:** Powder×1 vial, can be stored at 4℃.

**Standard:** 10 nmol/ml tetraethoxypropane, 5ml×1 bottle, can be stored at 4℃.

**Microscale Reagent 3 preparation in microscale operation procedure:**

(a) Prepare MDA Reagent 3 according to normal operation procedure: Pour 1 vial of MDA Reagent 3 in flask, add 64ml* 90℃～100℃ hot double distilled water, dissolve completely (you can heat solution properly during dissolving). Add 60ml glacial acetic acid after cooling, mix sufficiently.

(b) Dilute prepared MDA Reagent 3 with 50%** glacial acetic acid at ratio of 2:1, consider solution volume according to you need.

For example, if you need 15ml Reagent 3, then you can prepare 10ml Reagent 3 according to procedure above, then add 5ml 50% glacial acetic acid, mix sufficiently. Prepared can be stored at 4℃ in fridge away from light (you should get glacial acetic acid***).

Note: * Double distilled water will bulge when it becomes hot, and evaporation can not be ignored during heating process. So you need to add 64ml hot double distilled water to make sure 60ml double distilled water (after cooling) is add in reaction system .

** Mix 50ml double distilled water & 50ml glacial acetic acid together in order to prepare 50% glacial acetic acid.

*** Glacial acetic acid is also named as ethanoic acid, you can buy it from pharmaceuticals company or medicament company generally. It is better to buy analytical pure (AR) ethanoic acid, CH3COOH>99%.

This kit can be stored by cold preservation for at least 1 year.

**②**  **50T/48S assay kit:**

**Reagent 1:** Solution, 10ml×1 bottle, can be stored at room temperature. (It may coagulate in cold days, so before use, please warm this bottle in water bath in order to increasing dissolving rate. When solution becomes limpid, then you can use it for assay.)

**Reagent 2:** Solution, 6ml×1 bottle. When use, add 340ml double distilled water in each bottle. Can be stored at 4℃. **Note: Never drop on your skin**.

**Reagent 3:** Powder×1 vial, can be stored at 4℃.

**Standard:** 10 nmol/ml tetraethoxypropane, 5ml×1 bottle, can be stored at 4℃.

**Microscale Reagent 3 preparation in microscale operation procedure:**

(a) Prepare MDA Reagent 3 according to normal operation procedure: Pour 1 vial of MDA Reagent 3 in flask, add 32ml* 90℃～100℃ hot double distilled water, dissolve completely (you can heat solution properly during dissolving). Add 30ml glacial acetic acid after cooling, mix sufficiently.

(b) Dilute prepared MDA Reagent 3 with 50%** glacial acetic acid at ratio of 2:1, how much you use, how much you make.

For example, if you need 15ml Reagent 3, then you can prepare 10ml Reagent 3 according to procedure above, then add 5ml 50% glacial acetic acid, mix sufficiently. Prepared can be stored at 4℃ in fridge away from light (you should get glacial acetic acid***).

Note: * Double distilled water will bulge when it becomes hot, and evaporation can not be ignored during heating process. So you need to add 32ml hot double distilled water to make sure 30ml double distilled water (after cooling) is add in reaction system .

** Mix 50ml double distilled water & 50ml glacial acetic acid together in order to prepare 50% glacial acetic acid.

*** Glacial acetic acid is also named as ethanoic acid, you can buy it from pharmaceuticals company or medicament company generally. It is better to buy analytical pure (AR) ethanoic acid, CH3COOH>99%.

This kit can be stored by cold preservation for at least 1 year.

1. **Microscale operation table:**

|  | **Standard tube** | **Blank tube** | **Sample tube** | **Contrast tube**** |
| --- | --- | --- | --- | --- |
| 10 nmol/ml standard (ml) | a* |  |  |  |
| Dehydrated alcohol（ml） |  | a* |  |  |
| Sample to assay（ml） |  |  | a* | a* |
| Reagent 1 (ml) | a* | a* | a* | a* |
| Mix sufficiently by shaking test-tube stand | | | | |
| Reagent 2 (ml) | 1.5 | 1.5 | 1.5 | 1.5 |
| Reagent 3 (ml) | 1.5 | 1.5 | 1.5 |  |
| 50% glacial acetic acid（ml） |  |  |  | 1.5 |
| Mix sufficiently by vortex, seal test tube by refreshing film, pierce a small hole by needle, place in 95℃ water bath for 40 minutes, cool in tap water, centrifugate at 3500~4000rpm for 10 minutes.(If you centrifugate at lower speed (<3000rpm), then please extend time in order to make sedimentation complete). Take supernatant***, transfer in cuvettes of 1cm light path, measure absorbances of all tubes at 532nm (adjust zero by double distilled water). | | | | |

**a* reflects volumes of sample, standard, dehydrated alcohol, Reagent 1, these four volumes should be equal to each other.** For example, if you take 0.1ml sample, then you should also take 0.1ml standard, dehydrated alcohol, Reagent 1; if you take 0.2ml sample, then you should also take 0.2ml standard, dehydrated alcohol, Reagent 1. Absorbance reflects direct proportion with sample volume, so result won’t be disturbed.

****Generally, it only need to make 1~2 standard tubes, blank tubes, contrast tubes. If there is no haemolysis or lipidemia in samples, then you can skip contrast tube, use blank tube to instead.**

*******Use micropipet to transfer supernatant in cuvette, please avoid transfer by pouring or sediments may enter cuvette and disturb absorbance.

**Note 1:** OD values measured by microscale method are higher than use normal method, but final results won’t be disturbed.

**Note 2:** For 5% homogenate, a*=0.1~0.2ml.

**Note 3:** If OD_Sample_ is too low, then you can extend water bath period from 40 minutes to 80 minutes. But if you do like this, then please extend all MDA assays’ water bath period to 80 minutes in order to avoid difference between batches.

**Note 4:** Referenced OD_Standard_ of this method:

When standard volume is 0.1ml,

ODStandard – ODBlank = 0.103~0.112

When standard volume is 0.2ml,

ODStandard – ODBlank = 0.206~0.224

**(4) Formulas:**

**①** **Blood serum (or plasma) MDA assay:**


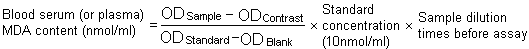


**②** **Tissue MDA assay:**


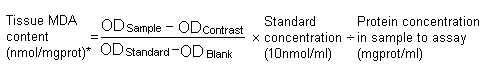


* nmol/mgprot means nanomole per protein in milligram

**(5) Examples:**

① Take 0.1ml human blood serum without dilution to measure MDA content, in results, OD_Sample_ is 0.030, OD_Standard_ is 0.104, OD_Blank_ is 0.009(use blank tube to instead contrast tube). Calculate as follows:


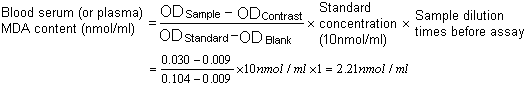


② Take 0.1ml 5% rat lung tissue homogenate to measure MDA content, in results, OD_Sample_ is 0.062, OD_Standard_ is 0.104, OD_Blank_ is 0.009 (use blank tube to instead contrast tube), protein concentration in 5% rat lung tissue homogenate is 2.45mgprot/ml. Calculate as follows:


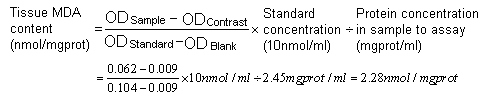
`

**8. Hyperlipidemia sample MDA assay procedure:**

This method can be used to measure MDA content in hyperlipidemia blood or lipids. For example, you can use this method to measure MDA content in soybean oil, salad oil or rubsen seed oil, etc.

1. **Slight hyperlipidemia blood MDA assay:**

Slight hyperlipidmia blood serum (or plasma) appears less limpid, you can also measure MDA according to common operation method or midrange hyperlipidemia operation method.

**① Operation table:**

|  | **Standard tube** | **Blank tube** | **Sample tube** | **Contrast tube**** |
| --- | --- | --- | --- | --- |
| 10 nmol/ml standard (ml) | a* |  |  |  |
| Dehydrated alcohol（ml） |  | a* |  |  |
| lipid sample or slight hyperlipidemiablood serum(or plasma)（ml） |  |  | a* | a* |
| Reagent 1 (ml) | a* | a* | a* | a* |
| Mix sufficiently by shaking test-tube stand | | | | |
| Reagent 2 (ml) | 3.0 | 3.0 | 3.0 | 3.0 |
| Reagent 3 (ml) | 1.0 | 1.0 | 1.0 |  |
| 50% glacial acetic acid（ml） |  |  |  | 1.0 |
| Mix sufficiently by vortex, seal test tube by refreshing film, pierce a small hole by needle, place in 95℃ water bath for 40 minutes, cool in tap water, centrifugate at 3500~4000rpm for 10 minutes.(If you centrifugate at lower speed (<3000rpm), then please extend time in order to make sedimentation complete). Take supernatant***, transfer in cuvettes of 1cm light path, measure absorbances of all tubes at 532nm (adjust zero by double distilled water). | | | | |

a* is sample volume, hyperlipidemia blood serum (or plasma) sample volume is 100μl, lipid sample volume is 30~50μl, volumes of standard, sample & Reagent 1 should be equal to each other.

****Generally, it only need to make 1~2 standard tubes, blank tubes, contrast tubes. If there is no haemolysis or lipidemia in samples, then you can skip contrast tube, use blank tube to instead.**

*******Use micropipet to transfer supernatant in cuvette, please avoid transfer by pouring or sediments may enter cuvette and disturb absorbance.

**②** **Referenced ODStandard:**

When standard volume is 0.1ml, OD_Standard_ – OD_Blank_ = 0.065~0.070

When standard volume is 0.2ml, OD_Standard_ – OD_Blank_ = 0.130~0.140

**③ Formula:**


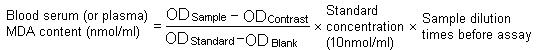


**④ Example:**

Take slight hyperlipidemia rabbit blood serum without dilution to measure MDA content, in results, OD_Sample_ is 0.080, OD_Standard_ is 0.046, OD_Blank_ is 0.010(use blank tube to instead contrast tube). Calculate as follows:


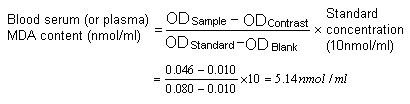


**(2) Midrange hyperlipidemia blood MDA assay:**

**Midrange hyperlipidmia blood serum (or plasma) appears quite turbid.**

**① Sample pretreatment:**

Dilute midrange hyperlipidmia sample with physiological saline at ratio of 1:2 or 1:3.

**② Operation table A:**

|  | **Standard tube** | **Blank tube** | **Sample tube** | **Contrast tube**** |
| --- | --- | --- | --- | --- |
| 10 nmol/ml standard (ml) | a* |  |  |  |
| Dehydrated alcohol（ml） |  | a* |  |  |
| Diluted midrange hyperlipidemia blood serum(or plasma（ml） |  |  | a* | a* |
| Reagent 1 (ml) | a* | a* | a* | a* |
| Mix sufficiently by shaking test-tube stand | | | | |
| Reagent 2 (ml) | 1.0 | 1.0 | 1.0 | 1.0 |
| Reagent 3 (ml) | 1.0 | 1.0 | 1.0 |  |
| 50% glacial acetic acid（ml） |  |  |  | 1.0 |
| Mix sufficiently by vortex, seal test tube by refreshing film, pierce a small hole by needle, place in 95℃ water bath for 80 minutes, cool in tap water, centrifugate at 3500~4000rpm for 10 minutes.(If you centrifugate at lower speed (<3000rpm), then please extend time in order to make sedimentation complete). Take supernatant***, transfer in cuvettes of 1cm light path, measure absorbances of all tubes at 532nm (adjust zero by double distilled water). | | | | |

a* is sample volume, volumes of standard, sample, dehydrated alcohol & Reagent 1 should be equal to each other. Sample can be diluted with physiological saline at ratio of 1:2 or 1:3. Sample volumes of standard, sample, dehydrated alcohol & Reagent 1 can be 0.1ml, 0.2ml or 0.3ml.

****Generally, it only needs to make 1~2 standard tubes, blank tubes, contrast tubes. If there is no haemolysis or lipidemia in samples, then you can skip contrast tube, use blank tube to instead.**

*******Use micropipet to transfer supernatant in cuvette, please avoid transfer by pouring or sediments may enter cuvette and disturb absorbance.

**③ Operation table B:**

|  | **Standard tube** | **Blank tube** | **Sample tube** | **Contrast tube**** |
| --- | --- | --- | --- | --- |
| 10 nmol/ml standard (ml) | a* |  |  |  |
| Dehydrated alcohol (ml) |  | a* |  |  |
| Midrange hyperlipidemia blood serum(or plasma (ml) |  |  | a* | a* |
| Physiological saline (ml) | 2a* | 2a* | 2a* | 2a* |
| Mix sufficiently by shaking test-tube stand | | | | |
| Reagent 1 (ml) | 4a* | 4a* | 4a* | 4a* |
| Mix sufficiently by shaking test-tube stand | | | | |
| Reagent 2 (ml) | 1.0 | 1.0 | 1.0 | 1.0 |
| Reagent 3 (ml) | 1.0 | 1.0 | 1.0 |  |
| 50% glacial acetic acid（ml） |  |  |  | 1.0 |
| Mix sufficiently by vortex, seal test tube by refreshing film, pierce a small hole by needle, place in 95℃ water bath for 80 minutes, cool in tap water, centrifugate at 3500~4000rpm for 10 minutes.(If you centrifugate at low speed (<3000rpm), then please extend time in order to make sedimentation complete). Take supernatant***, transfer in cuvettes of 1cm light path, measure absorbances of all tubes at 532nm (adjust zero by double distilled water). | | | | |

a* is sample volume, volumes of standard, sample & dehydrated alcohol should be equal to each other. Physiological saline has twice volume, Reagent 1 has 4 times volume. For example, if you take 0.1ml hyperlipidemia blood serum, then you should take 0.1ml standard, 0.1 dehydrated alcohol, 0.2ml physiological saline, 0.4ml Reagent 1.

****Generally, it only need to make 1~2 standard tubes, blank tubes, contrast tubes. If there is no haemolysis or lipidemia in samples, then you can skip contrast tube, use blank tube to instead.**

*******Use micropipet to transfer supernatant in cuvette, please avoid transfer by pouring or sediments may enter cuvette and disturb absorbance.

**④** **Referenced ODStandard:**

When standard volume is 0.1ml, OD_Standard_ – OD_Blank_ = 0.113~0.122

When standard volume is 0.2ml, OD_Standard_ – OD_Blank_ = 0.216~0.234

**⑤ Formula:**


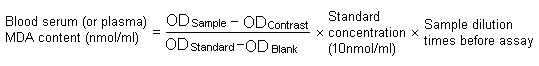


**⑥ Example:**

Take midrange hyperlipidemia rabbit blood serum without dilution to measure MDA content according to Operation table B, in results, OD_Sample_ is 0.124, OD_Standard_ is 0.101, OD_Blank_ is 0.010 (use blank tube to instead contrast tube). Calculate as follows


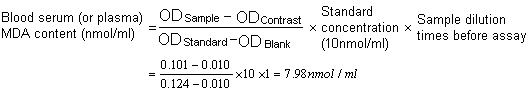


**(3) Heavy hyperlipidemia blood MDA assay:**

**Heavy hyperlipidemia blood serum (or plasma) appears like white milk.**

**① Operation table:**

|  | **Standard tube** | **Blank tube** | **Sample tube** | **Contrast tube**** |
| --- | --- | --- | --- | --- |
| 10 nmol/ml standard (ml) | a* |  |  |  |
| Dehydrated alcohol 1 (ml) |  | a* |  |  |
| lipid sample or hyperlipidemia blood serum (or plasma) (ml) |  |  | a* | a* |
| Dehydrated alcohol 2 (ml) | 2a* | 2a* | 2a* | 2a* |
| Extract sufficiently by mixing for 1 minute | | | | |
| Reagent 1 (ml) | 3a* | 3a* | 3a* | 3a* |
| Mix sufficiently by shaking test-tube stand | | | | |
| Reagent 2（ml） | 1.0 | 1.0 | 1.0 | 1.0 |
| Reagent 3（ml） | 1.0 | 1.0 | 1.0 |  |
| 50% glacial acetic acid（ml） |  |  |  | 1.0 |
| Mix sufficiently by vortex, seal test tube by refreshing film, pierce a small hole by needle, place in 95℃ water bath for 80 minutes, cool in tap water, centrifugate at 3500~4000rpm for 10 minutes.(If you centrifugate at lower speed (<3000rpm), then please extend time in order to make sedimentation complete). Take supernatant***, transfer in cuvettes of 1cm light path, measure absorbances of all tubes at 532nm (adjust zero by double distilled water). | | | | |

a* is sample volume, volumes of standard, dehydrated alcohol 1, sample should be equal to each other, dehydrated alcohol 2 has twice volume, Reagent 1 has 3 times volume. For example, if you take 0.1ml hyperlipidemia blood serum, then you should take 0.1ml standard, 0.1ml dehydrated alcohol 1, 0.2ml dehydrated alcohol 2, 0.3ml Reagent 1.

****Generally, it only need to make 1~2 standard tubes, blank tubes, contrast tubes. If there is no haemolysis or lipidemia in samples, then you can skip contrast tube, use blank tube to instead.**

*******Use micropipet to transfer supernatant in cuvette, please avoid transfer by pouring or sediments may enter cuvette and disturb absorbance.

**②** **Referenced OD_Standard_:**

When standard volume is 0.1ml, OD_Standard_ – OD_Blank_ = 0.114~0.123

When standard volume is 0.2ml, OD_Standard_ – OD_Blank_ = 0.216~0.235

**③ Formula:**


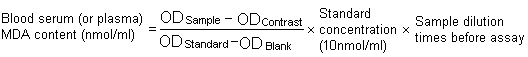


**④ Example:**

Take heavy hyperlipidemia rabbit blood serum without dilution to measure MDA content, in results, OD_Sample_ is 0.126, OD_Standard_ is 0.119, OD_Blank_ is 0.010 (use blank tube to instead contrast tube). Calculate as follows:

**
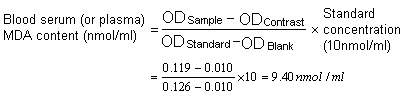
**

**9. Erythrocyte MDA assay procedure:**

1. **Microscale erythrocyte MDA assay procedure:**

**Please use the method when sample volume is quite small.**

**①** **Sample pretreatment:**

**You can use one of these two methods below:**

**a. Take whole blood by slide to measure MDA content:**

If you blood sample is very small & very precious, such as neonate finger blood or mouse tail blood, then you can add 1~2 droplets heparin on slide, mix sufficiently, dry it by stoving (<60℃) or blowing naturally. Add smallscaled blood sample on “heparin slide”, use micropipet to take heparin anticoagulated blood of volume you need, prepare 1:99 hemolysate.

**b. Take erythrocytes by glass microtainer and hemolysate preparation:**

(a) Take 20μl heparin anticoagulated whole blood by 20μl glass microtainer. Put glass microtainer at horizontal position, remove suction bulb quickly, put the terminal with longer vacant space in alcohol burner flame (at 1/3 site of flame, increase temperature until glass becomes red glow) until the terminal becomes globular clogging. Measure and record length of blood column (a cm). Pack microtainer by paper, insert microtainer in centrifuge tube, centrifugate at 1500rpm for 5~10 minutes. Take microtainer out of centrifuge tube, use small ;grinding wheel to carve at the dividing line of blood plasma and erythrocytes (you can also shear microtainer by scissors directly). Put microtainer with erythrocytes in a test tube with 1ml double distilled water (make sure microtainer’s opening is towards bottom of test tube, centrifugate again at 1500rpm for 5 minutes, now erythrocytes is at bottom of test tube. Remove microtainer by tweezers, put test tube on vortex mixer, mix sufficiently in order to make hemolysate.

(b) Blood dilution times calculation:


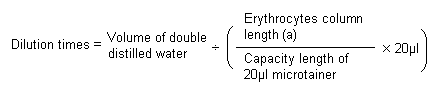


**② MDA assay operation procedure:**

**Method A (normal operation method)**

|  | **Standard tube** | **Blank tube** | **Sample tube** | **Contrast tube**** |
| --- | --- | --- | --- | --- |
| 10 nmol/ml standard (ml) | a* |  |  |  |
| Dehydrated alcohol 1 (ml) |  | a* |  |  |
| 1:x hemolysate (ml) |  |  | a* | a* |
| Reagent 1 (ml) | a* | a* | a* | a* |
| Mix sufficiently by shaking test-tube stand | | | | |
| Reagent 2 (ml) | 3 | 3 | 3 | 3 |
| Reagent 3 (ml) | 1 | 1 | 1 |  |
| 50% glacial acetic acid (ml) |  |  |  | 1 |
| Mix sufficiently by vortex, seal test tube by refreshing film, pierce a small hole by needle, place in 95℃ water bath for 40 minutes, cool in tap water, centrifugate at 3500~4000rpm for 10 minutes.(If you centrifugate at low speed (<3000rpm), then please extend time in order to make sedimentation complete). Take supernatant***, transfer in cuvettes of 1cm light path, measure absorbances of all tubes at 532nm (adjust zero by double distilled water). | | | | |

**a* reflects volumes of sample, standard, dehydrated alcohol, Reagent 1, these four volumes should be equal to each other.** For example, if you take 0.1ml sample, then you should also take 0.1ml standard, dehydrated alcohol, Reagent 1; if you take 0.2ml sample, then you should also take 0.2ml standard, dehydrated alcohol, Reagent 1. Absorbance reflects direct proportion with sample volume, so result won’t be disturbed.

****Generally, it only need to make 1~2 standard tubes, blank tubes, contrast tubes. If there is no haemolysis or lipidemia in samples, then you can skip contrast tube, use blank tube to instead.**

*******Use micropipet to transfer supernatant in cuvette, please avoid transfer by pouring or sediments may enter cuvette and disturb absorbance.

**Referenced OD_Standard_:**

When standard volume is 0.1ml, OD_Standard_ – OD_Blank_ = 0.065~0.130

When standard volume is 0.2ml, OD_Standard_ – OD_Blank_ = 0.130~0.140

**Note: When you use normal operation procedure or convenient operation procedure, if ODSample is too low, then you can extend water bath period from 40 minutes to 80 minutes. But if you do like this, then please extend all MDA assays’ water bath period to 80 minutes in order to avoid difference between batches.**

**Method B (microscale method)**

Results measured by this method are higher than using normal method, but final result won’t be disturbed.

**Please dilute prepared MDA Reagent 3 (using normal operation procedure) with 50% glacial acetic acid at ratio of 2:1, for example, you can dilute 100ml MDA Reagent 3 with 50ml 50% glacial acetic acid, mix sufficiently. You can also determine mixture volume by how mush you need, then you can start assay as follows:**

**(Note: To prepare 50% glacial acetic acid, pleaste mix 50ml double distilled water and 50ml glacial acetic acid.)**

|  | **Standard tube** | **Blank tube** | **Sample tube** | **Contrast tube**** |
| --- | --- | --- | --- | --- |
| 10 nmol/ml standard (ml) | a* |  |  |  |
| Dehydrated alcohol (ml) |  | a* |  |  |
| Sample to assay (ml) |  |  | a* | a* |
| Reagent 1 (ml) | a* | a* | a* | a* |
| Mix sufficiently by shaking test-tube stand | | | | |
| Reagent 2 (ml) | 1.5 | 1.5 | 1.5 | 1.5 |
| Reagent 3 (ml) | 1.5 | 1.5 | 1.5 |  |
| 50% glacial acetic acid (ml) |  |  |  | 1.5 |
| Mix sufficiently by vortex, seal test tube by refreshing film, pierce a small hole by needle, place in 95℃ water bath for 40 minutes, cool in tap water, centrifugate at 3500~4000rpm for 10 minutes.(If you centrifugate at lower speed (<3000rpm), then please extend time in order to make sedimentation complete). Take supernatant***, transfer in cuvettes of 1cm light path, measure absorbances of all tubes at 532nm (adjust zero by double distilled water). | | | | |

**a* reflects volumes of sample, standard, dehydrated alcohol, Reagent 1, these four volumes should be equal to each other.** For example, if you take 0.1ml sample, then you should also take 0.1ml standard, dehydrated alcohol, Reagent 1; if you take 0.2ml sample, then you should also take 0.2ml standard, dehydrated alcohol, Reagent 1. Absorbance reflects direct proportion with sample volume, so result won’t be disturbed.

****Generally, it only need to make 1~2 standard tubes, blank tubes, contrast tubes. If there is no haemolysis or lipidemia in samples, then you can skip contrast tube, use blank tube to instead.**

*******Use micropipet to transfer supernatant in cuvette, please avoid transfer by pouring or sediments may enter cuvette and disturb absorbance.

**Note: If OD_Sample_ is too low, then you can extend water bath period from 40 minutes to 80 minutes. But if you do like this, then please extend all MDA assays’ water bath period to 80 minutes in order to avoid difference between batches.**

**Referenced ODStandard:**

When standard volume is 0.1ml,OD_Standard_ – OD_Blank_ = 0.103~0.112

When standard volume is 0.2ml,OD_Standard_ – OD_Blank_ = 0.206~0.224

**③ Take hemolysate of certain concentration to do hemoglobinometry:**

**Hb measuring:** Take 1ml 100 times concentrated Hb assay stock solution (you can buy it from our institute), add 100ml water to make working solution. Take 20μl 1:x hemolysate, add 5ml Hb assay working solution, mix sufficiently, place quescently for 5 minutes, transfer in cuvettes of 1cm light path, measure and record absorbances of all tubes.

**Hb calculation:**

Hb content (gHb/L) = OD × 367.7

If your sample volume is very small, then you can halve sample and reagent (in fact, you can reduce volumes of sample and reagent as you want, but please keep the right ratio.

**④ Formula:**


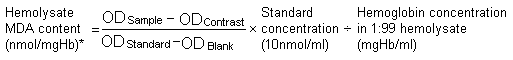


* nmol/mgHb means nanomole per milligram hemoglobin

**⑤ Example:**

a.Take 0.1ml 1:99 hemolysate to measure MDA content according to Method A. In results, OD_Sample_ is 0.064, OD_Standard_ is 0.077, OD_Blank_ is 0.009, OD_Contrast_ is 0.011.You can also use OD_Blank_ to instead of ODContrast, Hb content in 1:99 hemolysate is 11.08Hb/L. Calculate as follows:


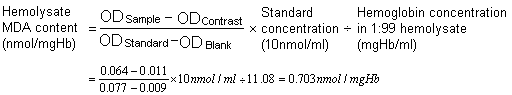


b.Take 0.1ml 1:99 hemolysate to measure MDA content. In results, ODSample is 0.084, OD_Standard_ is 0.100, OD_Blank_ is 0.015, OD_Contrast_ is 0.017.You can also use OD_Blank_ to instead of OD_Contrast_, Hb content in 1:99 hemolysate is 11.08Hb/L. Calculate as follows:


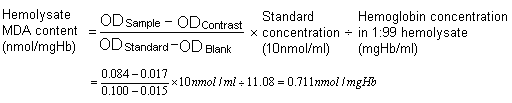


**(2) Erythrocyte MDA assay procedure in whole blood:**

**Please use the method when sample volume is large enough.**

**①** **Sample pretreatment:**

1. **Blood cell preparation:** Take 0.15ml heparin anticoagulated whole blood, add 2~3ml physiological saline, centrifugate at 2500~3000rpm for 5~10 minutes (mouse erythrocytes are fragile, so it is better to centrifugate at 1000~1500rpm for 5~10 minutes), remove supernatant and keep sediment of erythrocytes. Please remove supernatant completely or it will disturb results.
2. Hemolysate preparation: Take sediment after centrifugation, add double distilled water of 4 times whole blood volume (0.6ml), mix by vortex for 1 minute in order to lyze cells completely.
3. Extraction: Add dehydrated alcohol (or 95% alcohol) of 2 times whole blood, mix sufficiently by vortex for 30 seconds.
4. Protein sedimentation: add chloroform of twice whole blood volume (0.3ml), mix sufficiently by vortex for 1 minute, centrifugate at 3000~3500rpm for 5~10 minutes. Now liquid becomes 3 layers: upper layer is MDA extract solution, middle layer is hemoglobin sediment, underlayer is chloroform. Take supernatant and record volume (about 0.9ml).

**② Operation procedure:**

1. **1 nmol/ml standard working solution preparation:** Dilute 10nmol/ml standard 10 times, to say in other words, mix 1ml 10nmol/ml standard and 9ml dehydrated alcohol together in order to make 1nmol/ml standard solution.
2. **Operation table:**

**(If OD_Sample_ is too low, then you can extend water bath period from 40 minutes to 80 minutes. But if you do like this, then please extend all MDA assays’ water bath period to 80 minutes in order to avoid difference between batches.)**

|  | **Standard tube** | **Blank tube** | **Sample tube** |
| --- | --- | --- | --- |
| 1 nmol/ml standard（ml） | 0.8 |  |  |
| Dehydrated alcohol（ml） |  | 0.8 |  |
| Extract（ml） |  |  | 0.8 |
| Reagent 2（ml） | 1.0 | 1.0 | 1.0 |
| Reagent 3（ml） | 1.0 | 1.0 | 1.0 |
| Mix sufficiently by vortex, seal test tube by refreshing film, pierce a small hole by needle, place in 95℃ water bath for 40 minutes, cool in tap water, transfer in cuvettes of 1cm light path, measure absorbances of all tubes at 532nm (adjust zero by double distilled water). | | | |

**③ Formula:**


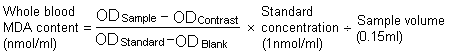


**④ Example:**

Take 0.15ml human whole blood, wash by physiological saline, centrifugate, remove supernatant, add 0.6ml double distilled water in sediment of erythrocytes, mix for 1 minute, add 0.3ml dehydrated alcohol, mix for 30 seconds, add 0.3ml chloroform, mix for 1 minute, centrifugate at 3500rpm for 8 minutes, take 0.8ml upperlayer extract to measure MDA. In results, OD_Standard_ is 0.112, OD_Sample_ is 0.038, OD_Blank_ is 0.012, calculate as follows:


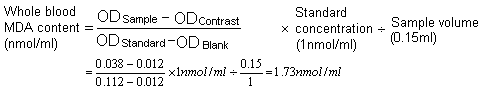


**11. Referenced MDA contents:**

● 1:99 human hemolysate: **6.38±0.072nmol/ml**

● Human blood serum (or plasma): **4.06±0.6nmol/ml**

● 10% rat brain homogenate: **4.72±1.44nmol/mgprot**

● 10% rat kidney homogenate: **2.70±0.46nmol/mgprot**

● 10% rat lung homogenate: **1.65±0.44nmol/mgprot**

● 10% rat cardiac muscle homogenate: **1.63±0.36nmol/mgprot**

● Mouse blood serum (or plasma): **5.30±0.73nmol/ml**

● 10% mouse brain homogenate: **16.3±5.04nmol/mgprot**

● 5% mouse cardiac muscle homogenate: **14.64±2.68nmol/mgprot**

● 10% mouse muscle homogenate: **51.59±11.08nmol/mgprot**

● 10% mouse liver homogenate: **8.26±2.75 nmol/mgprot**

**11. Announcements:**

(1) Please scrub test tubes clean and tidy, it is especially important when you measure microscale samples.

(2) Please mix sufficienly when you prepare reagents. First tip of reagent during measuring should be discarded, add samples and reagents vertically, don’t add on tube inner surface. Mix sufficiently before 95℃ water bath.

(3) Reagent 1 may coagulate in cold days, so before use, please warm this bottle in water bath in order to increasing dissolving rate. When solution becomes limpid, then you can use it for assay.

(4) Water bath’s time length and temperature should be stable.

(5) Centrifuging sedimentation must be completed, or it will disturb absorbance and lead to unstable result. In this situation, you can increase centrifuge speed (>3000rpm) or extend centrifuging period to make sedimentation completed.

(6) Use micropipet to transfer supernatant in cuvette, please avoid transfer by pouring or sediments may enter cuvette and disturb absorbance.

(7) In winter, if solution to assay appears “fog-like”, then you can put it in water bath and heat it slightly. When solution becomes limpid, transfer solution into cuvette by micropipet. If the solution keeps fog-like, then you should consider it may be hyperlipidemia.

(8) If it is hyperlipidemia blood serum or lipid samples, then you can treat this sample by adding dehydrated alcohol of equal volume. After treatment, you can start measurement according to procedures above.

(9) Sample volume: If you have large amount of sample, then you can double sample volume, during extraction, volumes of double distilled water, dehydrated alcohol & chloroform should be doubled. If your sample is anaemia blood sample, then you should double sample volume, but during extraction, volumes of double distilled water, dehydrated alcohol & chloroform should be constant.

(10) When wash erythrocytes, please remove supernatant as complete as possible in order to make extract volume accurate.

(11) It is better to use test tubes with stopper in 95℃ water bath in order to avoid reaction solution evaporation. If you want to use test tubes without stopper, then please seal test tube by refreshing film (tight by rubber band), pierce a small hole by needle to instead of stopper.

**12. Advantages:**

(1) All reagents of this kit are without stimulatory odors, they are unharmful for operators.

(2) Fast, accurate & convenient, 100 samples can be measured in 1 hour.

(3) High sensitivity, only need 0.1ml blood serum (or plasma) or even less.

(4) High repeatability, coefficient of variation=1.5%, there are only very small differences between several measurements of one sample.

(5) High chromatic stability, absorbance keeps stable in 24 hours after chromatic reaction.

(6) Reagents can be stored more than 1 year.

(7) Blood serum sample can be at 4℃ for 3~5 days or at -20℃ for 3 months~1 year.

(8) Wide application range: blood serum (or plasma), various kinds of tissue homogenates, cultured cells, etc.

(9) Major raw materals are imported, but their prices are midrange.

(10) Do not require expensive or special equipments, only need thermostatic water bath & spectrophotometer such as Type 721, 722, 751, 752.

(11) Insensitive for external factors such as temperature, it is stablest domestic MDA assay method now.

**Attachment: Experimental Methodology of sample pretreatments**
