# Supplementary material for: Low androgen levels induce ferroptosis of rat penile cavernous endothelial cells
Source: Sex Med. 2023 Aug 4;11(4):qfad043. doi: 10.1093/sexmed/qfad043 (PMC10401903; doi:10.1093/sexmed/qfad043)
Supplement: A006-2_GSH_Assay_Kit_Instruction_qfad043 [file a006-2_gsh_assay_kit_instruction_qfad043.doc]

**Microscale Reduced Glutathione assay kit**

**（Serial No.:A006-2 Microscale enzyme labelled method）**

**1. Reagent composition & preparation (96T)**

**Reagent 1:** Precipitant, 20ml×1 bottle, can be stored at room temperature for 6 month. This solution is supersaturared, if crystals seed out, then take supernatant for experiment.

**Reagent 2:** Buffer, 20ml×1 bottle, can be stored at 2～8℃ for 6 months.

**Reagent 3:** Chromogenic agent, 5ml×1 bottle, can be stored at 2～8℃ away from light for 6 months.

**Reagent 4:** GSH standard (Standard powder, 3.07mg×3 vials; Standard solvent stock solution, 10 ml×1 bottle), can be stored at 2～8℃ for 6 months.

**GSH standard solvent working solution preparation:** When use, dilute standard solvent stock solution with double distilled water at ratio of 1:9, consider solution volume according to your need, working solution should be used soon after preparation.

**1mmol/L GSH standard solution preparation:** GSH’s molecular weight is 307, add 3.07mg GSH standard powder in 10ml GSH standard solvent working solution, mix sufficiently, can be stored at 2℃～8℃ for 48 hours.

**20μmol/L GSH standard solution preparation:** Transfer 0.2ml 1mmol/L GSH standard solution in 9.8ml GSH standard solvent working solution. please use this solution soon after preparation.

**2. Sample pretreatment**

**(1) Cultured cells:** Rinse collected cells by PBS 1~2 times, do low speed centrifugation to get cell sediment, add 0.3～0.5ml 0.1M isotonic PBS (pH7.4) to make cell suspension, disrupt cells by ultrasonication or hand-driven.

**Supernatant preparation**:Take cracked cell suspension 0.1ml,and then add 0.1ml Reagent 1,mix well.and then centrifugate at 3500rpm for 10 minutes, take supernatant for assay.

**(2) Tissues:** Weigh sample accurately, add 9 times volume physiological saline according to mass (g)-volume(ml) ratio of 1:9, make homogenate in ice water bath, centrifugate at 2500rpm for 10 minutes, take supernatant for assay.

**Supernatant preparation**:Take tissue homogenate 0.1ml,and then add 0.1ml Reagent 1,mix well.and then centrifugate at 3500rpm for 10 minutes, take supernatant for assay.

(3) **Serum、blood plasma**：Take 0.05ml serum (blood plasma), add 0.2ml Reagent 1 working solution, mix sufficiently, centrifugate at 3500~4000rpm for 10 minutes. Take 1ml supernatant forassay.

(4) **Whole blood**：

**10% hemolysate preparation**: Take 0.1ml heparin anticoagulated whole blood, add 0.9ml double distilled water, mix sufficiently until hemolysate becomes limpid.

**Supernatant preparation**: Take 10% hemolysate 0.05ml,and then add 0.2ml Reagent 1,mix well.and then centrifugate at 3500rpm for 10 minutes, take supernatant for assay.

**3. Operation procedure:**

|  | Blank tube | Standard tube | Sample tube |
| --- | --- | --- | --- |
| Reagent 1(μl) | 100 |  |  |
| 20μmol/L GSH standard (ml) |  | 100 |  |
| Supernatant (μl） |  |  | 100 |
| Reagent 2 (μl) | 100 | 100 | 100 |
| Reagent 3 (μl) | 25 | 25 | 25 |
| Mix sufficiently, place for 5 minutes, transferto microplate measure OD values at 405nm | | | |

**4、Fomula：**

**Cell、Tissue：**


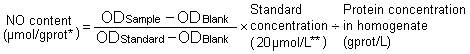


GSH

**5、Standard Curve：（The sensibility is1.5μmol/L）**

Dilute 1mmol/L GSH standard solution to：100μmol/L、50μmol/L、20μmol/L、10μmol/L、5μmol/L、0μmol/L to assay for Standard curve.

**6. Assay principle:**

Dithio-dinitrobenzoic acid can react with sulfhydryl compounds to produce a yellow compound, it can be quantitative estimated by colorimetric method at 405nm.

**7. Assay significance:**

Glutathione (GSH) is a low molecular mundificant which can get rid of O2-, H2O2 & LOOH. GSH is a tripeptide composed by glutamic acid, glycine & cysteine, it is main non-protein sulfhydryl compound in tissue and substrate of GSH-PX & GST (GSH-PX & GST need GSH to decompose hydroperoxides). GSH can also stabilize sulfhydryl containing enzymes and avoid oxidative damage of hemoglobin & other cofactors. Recently, GSH is proved to participate function of recovering vitamin E to reduction form. Absence or exhausting of GSH will induct various chemicals or environmental factors to cause or aggratate toxication, it may relate to increasing oxidative damage, so GSH content is an important factor to measure oxidizing ability in vivo.
