# Supplementary material for: Low androgen levels induce ferroptosis of rat penile cavernous endothelial cells
Source: Sex Med. 2023 Aug 4;11(4):qfad043. doi: 10.1093/sexmed/qfad043 (PMC10401903; doi:10.1093/sexmed/qfad043)
Supplement: A012-1_NO_Nitrate_Reductase_Assay_Kit_Instruction_qfad043 [file a012-1_no_nitrate_reductase_assay_kit_instruction_qfad043.doc]

**Nitric Oxide (NO) Assay Kit**

(Nitrate Reductase Method)

Serial No: A012

**1. Assay significance:**

NO has active chemical property, it is converted to nitrate (NO^3-^) & nitrite (NO^2-^). As result, it needs amount of blood serum NO^3-^ content plus blood serum NO^2-^ content to express NO level in vivo accurately. Some domestic units use metal cadmium (Cd) reduction method, but this method has tedious operations (protein in blood serum must be removed), low-controllable reactions (cadmium may reduce NO^2-^ further), low determinacy (cadmium can not reduce NO^3-^ to NO^2-^ completely), low accuracy.

Compared with metal cadmium method, nitrate reductase method is a sensitive, convenient, fast, stable, easy-generalized method.

**2. Assay principle:**

NO has active chemical property, it is converted to nitrate (NO^3-^) & nitrite (NO^2-^), NO^2-^ can also convert to NO^3-^. The method use nitrate reductase to reduce NO^3-^ to NO^2-^ selectively, calculate NO content by measuring OD values.

**3. Reagents compositon & preparation**

**（1）50T/48S:**

**Reagent 1:** Solution, 6ml×2 bottles, can be stored at -20℃ or even colder for 3 months. Please place bottle in 37℃ or at room temperature to dissolve completely before use.

**Reagent 2:** Solution, 6ml×2 bottles, can be stored at -20℃ or even colder for 3 months. Please place bottle in 37℃ or at room temperature to dissolve completely before use.

**Mixed reagent preparation:** Mix Reagent 1 and Reagent 2 at ratio of 1:1, how much you need, how much you prepare. After mixing completely, mixed reagent is available in 24 hours.

**Reagent 3:** Solution, 12ml×1 bottle, can be stored at room temperature for 6 months.

**Reagent 4:** Solution, 6ml×1 bottle, can be stored at room temperature for 6 months.

**Reagent 5:**Powder×1 vial, when use, add 20ml* 90℃～100℃ hot distilled water to dissolve completely, can be stored away from light.

**Reagent 6:** Powder×1 vial, when use, add 8ml double distilled water to dissolve it. Can be stored by cold preservation away from light. If its color becomes dark brown, then please discard it.

**Reagent 7:** Solution, 8ml×1 bottle, can be stored at room temperature for 6 months.

**Chromogenic agent:** How much you need, how much you make (if you can exhaust chromogenic agent in 1 month, then you can prepare it in 1 time). Mix Reagent 5, Reagent 6 & Reagent 7 at ratio of 2.5:1:1. Can be stored in 40ml clean square bottle away from light. There are some crystals seed out in cold days, so when you use it again, please place it in 100℃ water bath, dissolve completely by shaking repeatedly.

**10mmol/L standard:** 0.5ml×1 vial, can be stored at -20℃ or even colder for 6 months.

**100μmol/L standard working solution preparation:** Take 0.1ml standard, add double distilled water until volume reaches to 10ml (100 times dilution), mix sufficiently, 100 μmol/L standard working solution is prepared. This working solution should be used soon after preparation.

**Attachment:** Double distilled water for Reagent 5, Reagent 6 and standard’s preparations: 40ml×2 bottles.

**(2)** **25T/24S:**

**Reagent 1:** Solution, 6ml×1 bottle, can be stored at -20℃ or even colder for 3 months. Please place bottle in 37℃ or at room temperature to dissolve completely before use.

**Reagent 2:** Solution, 6ml×1 bottle, can be stored at -20℃ or even colder for 3 months. Please place bottle in 37℃ or at room temperature to dissolve completely before use.

**Mixed reagent preparation:** Mix Reagent 1 and Reagent 2 at ratio of 1:1, how much you need, how much you prepare. After mixing completely, mixed reagent is available in 24 hours.

**Reagent 3:** Solution, 6ml×1 bottle, can be stored at room temperature for 6 months.

**Reagent 4:** Solution, 3ml×1 bottle, can be stored at room temperature for 6 months.

**Reagent 5:**Powder×1 vial, when use, add 10ml* 90℃～100℃ hot distilled water to dissolve completely, can be stored away from light.

**Reagent 6:** Powder×1 vial, when use, add 4ml double distilled water to dissolve it. Can be stored by cold preservation away from light. If its color becomes dark brown, then please discard it.

**Reagent 7:** Solution, 4ml×1 bottle, can be stored at room temperature for 6 months.

**Chromogenic agent:** How much you need, how much you make (if you can exhaust chromogenic agent in 1 month, then you can prepare it in 1 time). Mix Reagent 5, Reagent 6 & Reagent 7 at ratio of 2.5:1:1. Can be stored in 40ml clean square bottle away from light. There are some crystals seed out in cold days, so when you use it again, please place it in 100℃ water bath, dissolve completely by shaking repeatedly.

**10mmol/L standard:** 0.5ml×1 vial, can be stored at -20℃ or even colder for 6 months.

**100μmol/L standard working solution preparation:** Take 0.1ml standard, add double distilled water until volume reaches to 10ml (100 times dilution), mix sufficiently, 100 μmol/L standard working solution is prepared. This working solution should be used soon after preparation.

**Attachment:**Double distilled water for Reagent 5, Reagent 6 and standard’s preparations: 40ml×1 bottle.

**Note:** * Reagent 5 is supersaturated solution, it is better to add 11.5ml hot distilled water (consider thermal expansion), adding, heating by water bath & stirring by glass rod in same time to dissolve completely. There may be crystals in old Reagent 5 (from last experiment), please heat and stir it to dissolve completely.

**4. Operation procedures**

**（1）Liquid sample** (such as blood serum, gastric juice, urine, cell culture fluid, etc.) NO assay:

**①** **Pretreatment:** According to Experimental Methodology.

**②** **Operation table:**

|  | **Blank tube** | **Standard tube** | **Sample tube** |
| --- | --- | --- | --- |
| **Double distilled (ml)** | 0.1 |  |  |
| **100μmol/L standard**  **working solution (ml)** |  | 0.1 |  |
| **Liquid sample (ml)** |  |  | 0.1 |
| **Mixed reagent (ml)** | 0.4 | 0.4 | 0.4 |
| Mix sufficiently, place in 37℃ water bath for 60 minutes accurately | | | |
| **Reagent 3 (ml)** | 0.2 | 0.2 | 0.2 |
| **Reagent 4 (ml)** | 0.1 | 0.1 | 0.1 |
| Mix sufficiently by vortex for 30 seconds, place quescently for 40 minutes, centrifugate at 3500~4000 rpm for 10 minutes, take supernatant for chromogenic reaction. | | | |
| **Supernatant (ml)** | 0.5 | 0.5 | 0.5 |
| **Chromogenic agent (ml)** | 0.6 | 0.6 | 0.6 |
| Mix sufficiently, place quescently for 10 minutes, transfer in cuvettes of 0.5cm light path, measure OD values of all tubes at 550nm (adjust zero by distilled water) | | | |

**Note:** a. If you do this assay in winter, then please prewarm all reagents at 37℃ for 5 minutes.

b. If you have not enough supernatant, please extend centrifugation time length or take less supernatant (such as 0.4ml or 0.45ml), but please make sure that all tubes in one batch have sample sample volume. Never add sediment in tubes.

**③ Formula:**


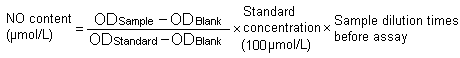


**④ Examples:**

a. Take 0.1ml undiluted chicken blood serum to measure NO content, in results, OD_Blank_ is 0.080, OD_Standard_ is 0.169, OD_Sample_ is 0.109. Calculate as follows:


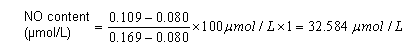


b. Take 0.1ml rat cerebrospinal fluid to measure NO content, in results, ODBlank is 0.077, ODStandard is 0.172, ODSample is 0.093. Calculate as follows:


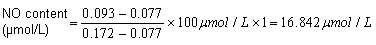


**(2) Tissue sample NO assay**

**①** **Pretreatment:** According to Experimental Methodology.

**②** **Operation table:**

|  | **Blank tube** | **Standard tube** | **Sample tube** |
| --- | --- | --- | --- |
| **Double distilled (ml)** | 0.5 |  |  |
| **100μmol/L standard**  **working solution (ml)** |  | 0.5 |  |
| **Liquid sample (ml)** |  |  | 0.5 |
| **Mixed reagent (ml)** | 0.4 | 0.4 | 0.4 |
| Mix sufficiently, place in 37℃ water bath for 60 minutes accurately | | | |
| **Reagent 3 (ml)** | 0.2 | 0.2 | 0.2 |
| **Reagent 4 (ml)** | 0.1 | 0.1 | 0.1 |
| Mix sufficiently by vortex for 30 seconds, place quescently for 40 minutes, centrifugate at 3500~4000 rpm for 10 minutes, take supernatant for chromogenic reaction. | | | |
| **Supernatant (ml)** | 0.8 | 0.8 | 0.8 |
| **Chromogenic agent (ml)** | 0.6 | 0.6 | 0.6 |
| Mix sufficiently, place quescently for 10 minutes, transfer in cuvettes of 0.5cm light path, measure OD values of all tubes at 550nm (adjust zero by distilled water) | | | |

**Note:** a. If you do this assay in winter, then please prewarm all reagents at 37℃ for 5 minutes.

b. If you have not enough supernatant, please extend centrifugation time length or take less supernatant (such as 0.4ml or 0.45ml), but please make sure that all tubes in one batch have sample sample volume. Never add sediment in tubes.

**③ Formula:**


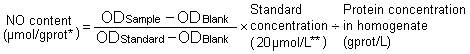


**Note: *** μmol/gprot means micromole per gram protein

** 0.1ml 100μmol/L standard working solution is diluted with 0.4ml double distilled water, it equals to 5 times dilution, standard concentration here is 20μmol/L.

**④ Examples:**

a. Take 0.5ml 10% mouse brain tissue measure NO content, in results, ODBlank is 0.071, ODStandard is 0.156, ODSample is 0.096, protein concentration in 10% mouse brain tissue homogenate supernatant is 4.249gprot/L. Calculate as follows:


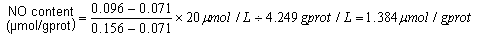


b. Take 0.5ml 10% Acipenser sinensis brain tissue measure NO content, in results, ODBlank is 0.070, ODStandard is 0.153, ODSample is 0.132, protein concentration in 10% Acipenser sinensis brain tissue homogenate supernatant is 4.533gprot/L. Calculate as follows:


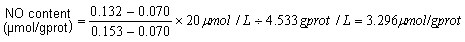


**5. Referenced values**

**(1) Optimal sampling volumes:**

● Bovine blood serum: 300μl ● Sheep blood serum: 300μl

● Rat & mouse blood serum: 100μl ● Rabbit blood serum: 100μl

● 10% tissue homogenate: 500μl ● Cell suspension: 500μl ● Cell culture fluid: 100μl

**(2)** **Normal NO contents:**

| **Sample** | **Referenced sampling volume** | **Referenced NO content** |
| --- | --- | --- |
| Rat blood serum | 100μl | 38.0±23.4μmol/L |
| Mouse blood serum | 100μl | 49.96±23.76μmol/L |
| Rabbit blood serum | 100μl | 99.16±39.5μmol/L |
| Dog blood serum | 100μl | 89.51±32.13μmol/L |

**6. Announcements**

(1) NO content is always low in tissue sample, so homogenates are 10% or 20% generally, centrafugation speed is 2000 rpm for 10 minutes (Do not use higher rotate speed or longer centrifugation time), Sampling volume is relatively large, it can be 0.3~0.5ml properly.

(2) Please avoid free-thawing Reagent 1, Reagent 2 & standard repeatedly. If you want to do experiments in batches (>3), then you can subpack and store Reagent 1, Reagent 2 & standard when you do first experiment, when you do other experiment, take this reagents according to how much you use. Prepared chromogenic agent should be stored away from light.

(3) If you don’t use samples (such as blood serum, blood plasma, tissues, etc.) immediately, then you can store them at -70℃ or even lower temperature. Deepfreezed samples are available in half year.

(4) All reagent preparations and blank tubes need NO^2-^-free double or triple distilled water (This kit includes double distilled water).

(5) After complete reaction and centrifugation, please avoid suck sediment when you take supernatant, or OD values will increase largely, validity of results will be disturbed seriously

(6) Test tube selection:

a. It is suggested to use disposable plastic test tubes (preferably disposable plastic test tubes from our institute).

b. If you want to use glass test tubes, then please soak them in detergent solution for more than half hour, boil for 0.5~1 hour, brush carefully, rinse by tap water for 15~20 times, discard water, rinse by distilled water for 1~2 times, oven dry them.
