# Supplementary material for: Low androgen levels induce ferroptosis of rat penile cavernous endothelial cells
Source: Sex Med. 2023 Aug 4;11(4):qfad043. doi: 10.1093/sexmed/qfad043 (PMC10401903; doi:10.1093/sexmed/qfad043)
Supplement: A061-1_Total-GSH_Assay_Kit_Instruction_qfad043 [file a061-1_total-gsh_assay_kit_instruction_qfad043.docx]

Total-Glutathione (GSH) Assay Kit Instruction

## Principle of Measurement

The total-GSH or oxidized GSH (Glutathione disulfide, GSSG for short) content of tissues or body fluid is determined through the reversible reaction with 5,5’-dithiobis(2-nitrobenzoic acid) (DTNB).

## Reagent Composition and Preparation

|  | **Composition** | **48T** | **96T** | **Storage** |
| --- | --- | --- | --- | --- |
| **Reagent I** | Substrate I Powder | 1 Bottles | 2 Bottles | 4℃ |
|  | Buffer Solution | 1 Bottle×5ml | 2 Bottles×5ml | 4℃ |
| Preparation of **Reagent I Solution**: Dilute each one bottle powder with 5ml buffer solution and dissolve thoroughly. The solution should be preserved at 4℃ | | | | |
| **Reagent II** | Substrate II Stock | 25μl×1 tube | 25μl×2 tubes | -20℃ |
|  | Diluent | 500μl×tube | 500μl×2 tubes | -20℃ |
| Preparation of **Reagent II Solution**: Dilute the stock with diluent at the ratio 1:19. Prepare the solution prior to use with the amount needed. | | | | |
| **Reagent III** | Powder | Powder×3 tubes | Powder×5 tubes | -20℃ |
|  | Diluention | 1ml×3 tubes | 1ml×5 tubes | 4℃ |
| Preparation of **Reagent III Solution**: Dissolve the powder with 1ml diluent. Prepare the solution prior to use with the amount needed. | | | | |
| **Reagent IV** | Powder | Powder×3 bottles | Powder×5 bottles | 4℃ |
| **Preparation of Reagent IV:** Dissolve each bottle of powder with boiled double distilled water (DDW) to 10ml and cool the solution. The solution can be preserved at 4℃for 3 days. | | | | |
| **Reagent V** | Stock Solution | 1 Bottle×15μl | 1 Bottle×30μl | 4℃ |
|  | Reagent V Solvent | 1 Bottle×150μl | 1 Bottle×300μl | 4℃ |
| **Preparation of Reagent V**: Prior to use, dilute the stock solution with solvent at the ratio 1:9. | | | | |
| **Reagent VI** | Solution | 1 Bottle×250μl | 1 Bottle×500μl | 4℃ |
| **Note**: Reagent VI is viscoid and be patient and careful when extracting the solution. | | | | |
| **GSSG Standard** | 3.07mg | Powder×1 tube | Powder×1 tube | 4℃ |
| Preparation of **1mM GSSG Stock Solution:** Dissolve the powder with 10ml DDW and the solution is stable at -20℃ for a month. | | | | |
| Preparation of 50μM GSSG Standard Solution: Dilute the 1mM solution to 20 times the initial volume. Prepare before use. | | | | |
| **GSH Standard** | 3.07mg | Powder×1 tube | Powder×1 tube | 4℃ |
| Preparation of **1mM GSH Stock Solution:** Dissolve the powder with 10ml DDW and the solution is stable at -20℃ for a month. | | | | |
| Preparation of **50μM GSH Standard Solution**: Dilute the 1mM solution to 20 times the initial volume. Prepare before use. | | | | |

## Sample Pretreatment

1. **Whole Blood Sample**
2. Take blood and add Heparin or EDTA for anticoagulation.
3. Extract 100 μl blood sample and add 400 μl freshly prepared reagent IV solution with the dilute coefficient of 5. Vortex for 30 s and then set aside the sample at 4°C for 5 min.
4. Centrifuge at 3,500 rpm for 10 min and extract the supernatant at 4°C. The supernatant should be placed under -20°C for preservation.
5. **Red Blood Cell Sample**
6. Take blood and add Heparin or EDTA for anticoagulation.
7. Centrifuge at 2,000 rpm for 10 min and carefully remove the upper plasma and the white blood cell layer on the surface of red blood cell layer.
8. Extract 100 μl red blood cell and add 400 μl freshly prepared reagent IV solution with the dilute coefficient of 5. Vortex for 30 s and then set aside the sample at 4°C for 5 min.
9. Centrifuge at 3,500 rpm for 10 min and extract the supernatant at 4°C. The supernatant should be placed under -20°C for preservation.
10. **Serum or Plasma Sample**
11. Take blood and add Heparin or EDTA for anticoagulation.
12. Centrifuge at 2,000 rpm for 10 min and carefully extract the upper plasma layer.
13. Extract 100 μl plasma and add 400 μl freshly prepared reagent IV solution with the dilute coefficient of 5. Vortex for 30 s and then set aside the sample at 4°C for 5 min.
14. Centrifuge at 3,500 rpm for 10 min and extract the supernatant at 4°C. The supernatant should be placed under -20°C for preservation.
15. **Tissue Sample**
16. Rinse the freshly prepared tissues with saline and remove the excess water on tissues.
17. Weigh the tissues and add reagent IV with the ratio of 1:4(g/ml) and homogenize in an ice water bath.
18. Centrifuge the homogenate at 3,500rpm for 10 min and extract the supernatant at 4°C. The supernatant should be placed under -20°C for preservation.

## Procedures of Measurement

1. **T-GSH Measurement**

| **Compositions** | **Standard** | **Blank** |
| --- | --- | --- |
| 50 μM GSH Standard(μl) | 10 |  |
| Sample(μl) |  | 10 |
| Reagent I(μl) | 100 | 100 |
| Reagent II(μl) | 10 | 10 |
| Mix and after mixing, set aside at room temperature for 2 min | | |
| Reagent III(μl) | 50 | 50 |

Record the timing right after the addition of reagent III. Mix and extract the solution into microplate At 405nm, record the absorbance at 30 s (A_1_) and set aside for 5 min at room temperature. At 5 min 30 s, record the absorbance (A_2_) at the same wavelength.

1. **GSSG Measurement**
2. **Pre-Treatment**

| **Compositions** | **Standard** | **Sample** |
| --- | --- | --- |
| 50 μM GSSG Standard(μl) | 100 |  |
| Sample(μl) |  | 100 |
| Reagent V(μl) | 2 | 2 |
| Reagent VI(μl) | 5 | 5 |

Vortex for 1min and warm the solution at 37°C for 30 min for further use.

1. **GSSG Measurement**

| Compositions | Standard | Sample |
| --- | --- | --- |
| Standard Pretreatment(μl) | 10 |  |
| Sample Pretreatment(μl) |  | 10 |
| Reagent I(μl) | 100 | 100 |
| Reagent II(μl) | 10 | 10 |
| Mix and after mixing, set aside at room temperature for 2 min | | |
| Reagent III(μl) | 50 | 50 |

Record the timing right after the addition of reagent III. Mix and extract the solution into microplate. At 405nm, record the absorbance at 30 s (A_1_) and set aside for 5 min at room temperature. At 5 min 30 s, record the absorbance (A_2_) at the same wavelength.

**Note:** Pre-measurement can be done for 1-2 samples and in case high absorbance results obtained, dilute with reagent IV so that the T-GSH or GSSG concentration lays within the concentration range required for measurement.

## Standard Curve Establishment

1. **GSH Standard Curve**
2. Dissolve GSH in DDW to the concentration of 1mM as the standard solution and standard solution can be preserved at -20°C for a month.
3. Dilute the standard solution to 0mM (Blank), 0.0125mM, 0.025mM, 0.05mM and 0.1mM at 4°C for further use.
4. Procedures

| Compositions /μl | GSH Test Tube |
| --- | --- |
| GSH Solution with Different Concentrations(μl) | 10 |
| Reagent I(μl) | 100 |
| Reagent II(μl) | 10 |
| Mix and set aside the solution at room temperature for 2 min | |
| Reagent III(μl) | 50 |

Record the timing right after the addition of reagent III. Mix and extract the solution into microplate. At 405nm, record the absorbance at 30 s (A_1_) and set aside for 5 min at room temperature. At 5 min 30 s, record the absorbance (A_2_) at the same wavelength.

1. Results

| GSH Concentration | A_1_ | A_2_ | ΔA |
| --- | --- | --- | --- |
| 0μmol/L | 0.1686 | 0.1686 | 0.0000 |
| 12.5μmol/L | 0.1851 | 0.2173 | 0.0322 |
| 25μmol/L | 0.2005 | 0.2588 | 0.0583 |
| 50μmol/L | 0.2306 | 0.3374 | 0.1068 |
| 100μmol/L | 0.2722 | 0.4786 | 0.2064 |

1. Standard Curve


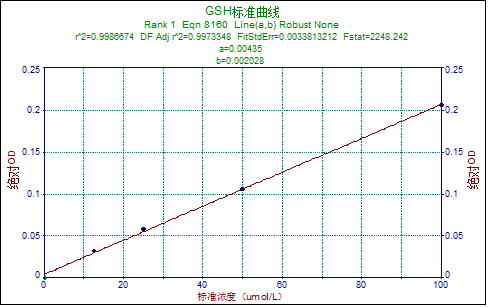


1. **GSSG Standard Curve**
2. Dissolve GSSG in DDW to the concentration of 1mM as the standard solution and standard solution can be preserved at -20°C for a month.
3. Dilute the standard solution to 0mM (Blank), 0.0125mM, 0.025mM, 0.05mM and 0.1mM at 4°C for further use.
4. Procedures
5. **Pre-treatment**

| Compositions | GSSG Test Tube |
| --- | --- |
| GSSG Solution with Different Concentrations（μl） | 100 |
| Reagent V（μl） | 2 |
| Reagent VI（μl） | 5 |

Vortex for 1min and warm the solution at 37°C for 30 min for further use.

1. GSSG Measurement

| Compositions | GSSG Test Tube |
| --- | --- |
| Pre-treated Solution with Different Concentrations（μl） | 10 |
| Reagent I（μl） | 100 |
| Reagent II（μl） | 10 |
| Mix and then set aside at room temperature for 2min | |
| Reagent III | 50 |

Record the timing right after the addition of reagent III. Mix and extract the solution into microplate. At 405nm, record the absorbance at 30 s (A_1_) and set aside for 5 min at room temperature. At 5 min 30 s, record the absorbance (A_2_) at the same wavelength.

1. Results

| GSH Concentration | A_1_ | A_2_ | ΔA |
| --- | --- | --- | --- |
| 0μmol/L | 0.1640 | 0.1640 | 0.0000 |
| 12.5μmol/L | 0.1720 | 0.2302 | 0.0582 |
| 25μmol/L | 0.1744 | 0.2799 | 0.1055 |
| 50μmol/L | 0.1799 | 0.3781 | 0.1982 |
| 100μmol/L | 0.1906 | 0.5679 | 0.3773 |

1. GSSG Standard Curve

## Calculation Formula

1. **Formula**

$$\begin{matrix} Total-GSH Content \\ (\mu mol/L ) \end{matrix}=\left. \frac{{\Delta A}_{Sample}{(A}_{2}-A_{1})}{{\Delta A}_{Standard}(A_{2}-A_{1})} \right.\times\begin{matrix} C_{Standard} \\ 50\mu M \end{matrix}\times\begin{matrix} Coefficient of Dilution \\ (Pretreatment Caused) \end{matrix}$$

$$\begin{matrix} GSSG Content \\ (\mu mol/L ) \end{matrix}=\left. \frac{{\Delta A}_{Sample}{(A}_{2}-A_{1})}{{\Delta A}_{Standard}(A_{2}-A_{1})} \right.\times\begin{matrix} C_{Standard} \\ 50\mu M \end{matrix}\times\begin{matrix} Coefficient of Dilution \\ (Pretreatment Caused) \end{matrix}$$

$$Reduced GSH Content=Total-GSH Content-2\times GSSG Content$$

2. **Standard Curve**
3. Find the corresponding T-GSH content with the standard curve established and multiply by the coefficient of dilution according to the steps of pre-treatment in order to achieve the T-GSH of the sample.
4. Find the corresponding GSSG content with the standard curve established and multiply by the coefficient of dilution according to the steps of pre-treatment in order to achieve the GSSG of the sample.

$Reduced GSH Content=Total-GSH Content-2\times GSSG Content$

## Note

1. For the fast rate of GSH metabolism, please treat the sample with reagent IV as soon as possible in order to lower the loss of GSH in sample.
2. The supernatant which is obtained via the treatment of samples with reagent IV can be preserved at -20°C for 6 months
3. The 5 minutes reaction time should be recorded precisely.

## Significance of Measurement

Researchers shows increasingly interest in Glutathione for its widespread existence and multifunction in vivo. It directly or indirectly functions in various biological phenomena and thus involved in numerous research topics such as mechanism of enzyme catalysis, synthesis of macromolecules like protein and DNA, metabolism, radiation, neoplasm, immunization, environmental toxins and ageing. Quite a lot of topics involving the GSH roles in cell defense against active oxygen or radicals. Among the samples measured, the GSH and GSSG content change in serum sample is a key indicator of oxide implicated in vivo.
